# Jointly modeling marine species to inform the effects of environmental change on an ecological community in the Northwest Atlantic

**DOI:** 10.1038/s41598-021-04110-0

**Published:** 2022-01-07

**Authors:** Sarah M. Roberts, Patrick N. Halpin, James S. Clark

**Affiliations:** 1 Nicholas School of the Environment, Duke University, Durham, NC 27708 USA; 2 Department of Statistical Science, Duke University, Durham, NC 27708 USA; 3 INRAE, 2 rue de la Papeterie, BP 76, 38402 Saint-Martin-d’Heres Cedex, France

**Keywords:** Marine biology, Climate-change ecology, Community ecology, Ecological modelling

## Abstract

Single species distribution models (SSDMs) are typically used to understand and predict the distribution and abundance of marine fish by fitting distribution models for each species independently to a combination of abiotic environmental variables. However, species abundances and distributions are influenced by abiotic environmental preferences as well as biotic dependencies such as interspecific competition and predation. When species interact, a joint species distribution model (JSDM) will allow for valid inference of environmental effects. We built a joint species distribution model of marine fish and invertebrates of the Northeast US Continental Shelf, providing evidence on species relationships with the environment as well as the likelihood of species to covary. Predictive performance is similar to SSDMs but the Bayesian joint modeling approach provides two main advantages over single species modeling: (1) the JSDM directly estimates the significance of environmental effects; and (2) predicted species richness accounts for species dependencies. An additional value of JSDMs is that the conditional prediction of species distributions can use not only the environmental associations of species, but also the presence and abundance of other species when forecasting future climatic associations.

## Introduction

Modeling and predicting the distribution and abundance of marine fish species is essential for effective fisheries management. Species distribution models must be sufficiently accurate to inform fisheries stock assessments. Single species distribution models (SSDMs) fit each species independently to a combination of abiotic environmental variables. However, species abundances and distributions are influenced by the abiotic environment as well as biotic interactions such as interspecific competition^1,2^ and predation^3^ that induce dependence between species. Proper treatment of biotic effects is needed for ecosystem-based fisheries management globally^4^, and in the Mid-Atlantic^5^ and greater North Atlantic^6^. Recent efforts to include the occurrence of other species as predictors or restricting the predicted distributions of species based on the distribution of another species^7^ do not provide valid inference on effects, because all species are encountered at random in samples—treating some species as fixed and others as random cannot be justified on probabilistic grounds. We present a joint species distribution model of the Northeast US. Large Marine Ecosystem (NEUS LME) that allows for environmental effects as well as dependence between species. We show that depth, temperature, and subregion have a strong influence on the community as a whole, and we identify environmental effects on rare species that we could not uncover with a single species model alone. By incorporating dependencies between species, we allow for conditional prediction, which accounts for the fact that certain biotic dependencies may be constraining distributions. Jointly modeling species together can help us more accurately identify how rare species will respond to changing conditions, the directionality and uncertainty of environmental effects, and which environmental variables are driving biomass for the entire community.

JSDMs can help to better inform the inter-specific dependencies that shape a species’ distribution and have been increasingly applied to marine studies in both univariate^8^ and multivariate studies^9,10^. By modeling species jointly, we can account for the fact that species do not respond independently of one another, in addition to environmental responses. Most marine fish species show indeterminate growth, and therefore biomass or count models are required to model species dynamics compared to presence absence models^11^. The Generalized Joint Attribute model (GJAM) allows for the joint responses with multiple observation types and zero inflation in fisheries data^12^ to better understand the potential effects of ensuing environmental change on the ecological community as a whole.

We use the NEUS LME to evaluate the joint distribution of a marine community in response to a warming environment. This area contains some of the most productive fisheries as well as the most rapidly increasing ocean temperatures that have been linked to shifts in the distribution of some fish species^13^ which has led to conflicts between regions over fisheries catch and management boundaries^14^. The long-term, scientifically collected NOAA/NEFSC trawl survey dataset has made this area of particular interest for researchers attempting to document shifting species distributions as a result of climate change and predicting further distribution shifts under projected ocean warming^13–16^. While most of this research relies on modeling the correlative relationship between single species and a suite of environmental variables and projecting distributions based on changing environmental conditions, these efforts omit the dependence between species.

The GJAM modeling framework allows us to evaluate species groups based on their combined responses to the environment and their residual correlation, i.e., the residual dependence between species after accounting for environmental effects in the mean structure of the model. Groupings include cold-water species such as Atlantic cod (*Gadus morhua*), haddock (*Melanogrammus aeglefinus*) and pollock (*Pollachius virens*) or warm water species such as summer flounder (*Paralichthys dentatus*) and smooth dogfish (*Mustelus canis*). The residual correlation further provides the opportunity to conditionally predict the responses of a set of species under different scenarios using the abundances of other species, which will ultimately help to inform how groups of species will react to changing environmental gradients on a more community-oriented level^17^. In terms of variable selection, GJAM allows for inverse prediction which comprehensively estimates the environmental importance for the entire community, by determining the capacity of the community to predict the environment. Thus, we select environmental variables that are well predicted by the community, and therefore explain important variation in many species. In general, this study is a critical first step at building a joint species distribution model of the NEUS LME that can be applied to ecosystem-based management, and more specifically, predicting joint distributions under climate change based on environmental variables and species co-dependence.

## Methods

### Species data

Species CPUE data were obtained from the National Oceanographic and Atmospheric Administration (NOAA) Northeast Fishery Science Center (NEFSC) U.S. NES bottom trawl survey, which, for almost 50 years, has collected abundance data for over 250 fish species in the spring and fall. The survey employs a stratified random design, with stations allocated proportionally to the stratum area. A 12 mm mesh coded liner is used to retain small-bodied and juvenile fish. All fish caught are weighed and counted^18^. We downloaded the data from OceanAdapt.com, which calibrates the CPUE for each species depending on survey ship. We cleaned the data for the years from 1998 to 2020, excluding years prior to 1997 due to many missing values for chlorophyll (Chl_a_). We only included strata that were consistently sampled in the spring and fall. To account for the seasonal migrations of many of the studied species, we modeled spring and fall seasons separately. We present the results for the fall CPUE, with the spring results and presence/absence results in the supplemental materials. We selected species that were present in at least 400 tows and with a biomass of at least 0.5 kg/tow (CPUE) in more than 100 tows. Finally, we removed roughly 400 tows per season with missing environmental covariates (see below). In the fall, we selected 30 species with 5217 observations, and in the spring, we selected 24 species with 5935 observations (see Supplemental Tables S1, S2).

### Environmental data

The study region includes Southern New England and The Gulf of Maine. We selected environmental covariates known to influence marine fish distributions and abundances. Depth, temperature (bottom and surface) and salinity (bottom and surface) were measured in situ during trawl surveys. Missing values were augmented with the data-assimilative HYbrid Coordinate Ocean Model (HYCOM) daily and then monthly data. HYCOM is an oceanographic model that produces 32 vertical layers including ocean temperature, salinity, sea surface height, and wind stress as well as other 3- and 4-dimensional variables. The system uses the Navy Coupled Ocean Data Assimilation (NCODA) system^19^ for data assimilation. NCODA uses the model forecast as a first guess in a multivariate optimal interpolation (MVOI) scheme and assimilates available satellite altimeter observations (along track obtained via the Naval Oceanographic Office Altimeter Data Fusion Center satellite) and in situ sea surface temperature as well as available in situ vertical temperature and salinity profiles from expendable bathythermographs, Argo floats, and moored buoys^20^. Seven HYCOM models (HYCOM + NCODA Global 1/12° Reanalysis GLBu0.08 Expts 19.0, 19.1, 90.9, 91.0, 91.1, 91.2) were temporally concatenated to create a continuous dataset of BT and salinity, ranging from 1992 to 2017. These model runs differed slightly in their configurations (time steps, advection scheme, mixing, vertical structure, slight change in NCODA, and MVOI transition to 3-dimensional analysis in 2013), but the differences are not expected to influence the applicability of the output^21^. The numbers of filled in missing values were 787 (7.0%) surface salinity (SSAL), 735 (6.5%) surface temperature (SST), 809 (7.2%) bottom temperature (BT), and 850 (7.6%) bottom salinity (BSAL). Chl_a_ was obtained from the MODIS satellite (monthly rasters from 2003 to 2019) on a monthly time step^22^, with missing values filled using the SeaWIFS satellite^23^ (1998 to 2009). Temperature, salinity and Chl_a_ data that were not collected in situ were downloaded using Google Earth Engine (HYCOM and MODIS)^24^. Benthic substrate (grain size in mm, referred to as SEDSIZE), subregion (Gulf of Maine or Southern New England), benthic land position (high, low, mid), and seabed form data (depression, high flat, high slope, low slope, mid flat, side slope, steep) were obtained from the Nature Conservancy’s Northwest Atlantic Marine Ecoregional Assessment^25^ (Supplemental Fig. S1).

### GJAM

To study the influence of the environmental covariates on the joint distribution of marine fish and invertebrate species we use the generalized joint attribute model (GJAM)^12^ and the corresponding R package (Version 2.5)^26^. Briefly, this multivariate Bayesian model allows us to jointly model the marine fish community and accounts for responses to the environment that can include combinations of continuous and discrete responses (e.g., CPUE and zeros) and the dependence between species. GJAM returns all parameters on the observation scale, in this case, CPUE and presence-absence. Products of model fitting include a species‐by‐species covariance matrix (Σ), species responses to predictor variables (**B**), and predicted responses. The species‐by‐species covariance matrix Σ captures residual codependence between species after removing the main structure explained by the model (also referred to as the residual correlation matrix). As a result, Σ allows for conditional prediction of some species under different scenarios for the abundances of others^27^.

CPUE is termed *continuous abundance* (CA) data in GJAM, meaning that observations are continuous with discrete zeros. Let *y*_*is*_ be the CPUE for species *s* at location *i*. For CA data GJAM expands the tobit model for (univariate) regression to the multivariate setting, where a latent variable *w*_*is*_ is equal to *y*_*is*_ when y_*is*_ is positive and zero otherwise,

1 $$y_{i,s}^{0} = \left\{ {\begin{array}{*{20}l} {w_{is} ,} \hfill & {w_{is} > 0\quad {\text{continuous}}} \hfill \\ {0,} \hfill & {w_{is} \le 0\quad {\text{discrete zero}}} \hfill \\ \end{array} } \right.$$

The length-*S* vector of all species responses **w**_*i*_ is continuous on the real line, and thus can be modeled with a multivariate normal. The model for **w**_*i*_ is

2 $$\begin{aligned} \left. {{\mathbf{w}}_{i} } \right|{\mathbf{x}}_{i, } {\mathbf{y}}_{i} & \sim \,MVN\left( {{\varvec{\mu}}_{i} ,{\Sigma }} \right) \times \mathop \prod \limits_{s = 1}^{S} {\mathcal{I}}_{is} \\ u_{{\varvec{i}}} & = {\mathbf{B}}^{\prime } {\mathbf{x}}_{{\varvec{i}}} \\ {\mathcal{I}}_{is} & = \mathop \prod \limits_{k \in C} I_{is,k}^{{I\left( {y_{is} = k} \right)}} \left( {1 - I_{is,k} } \right)^{{I\left( {y_{is} \ne k} \right)}} \\ \end{aligned}$$

$$\begin{aligned} {\mathcal{I}}_{is} & = I(w_{is} \le 0)^{{I\left( {y_{is} = 0} \right)}} \left[ {1 - I\left( {w_{is} \le 0} \right)} \right]^{{I\left( {y_{is} > 0} \right)}} \\ & \quad I(w_{is} > 0)^{{I\left( {y_{is} > 0} \right)}} \left[ {(1 - I(w_{is} > 0)} \right]^{{I\left( {y_{is} = 0} \right)}} \\ \end{aligned}$$

where the indicator function $I(\cdot )$ is equal to 1 when its argument is true and 0 otherwise. For presence-absence data, ${\mathbf{p}}_{{\varvec{i}}{\varvec{s}}}\boldsymbol{ }=\boldsymbol{ }\left(-\boldsymbol{\infty },\boldsymbol{ }0,\boldsymbol{ }\boldsymbol{\infty }\right).$ This is equivalent to Chib and Greenberg’s^28^ probit model which can be written as ${\mathcal{I}}_{is}=I({w}_{is}>{0)}^{I\left({y}_{is}>0\right)}I({w}_{is}\le {0)}^{1-{y}_{is}}$.

The mean vector ${\varvec{\mu}}_{i} = {\mathbf{B}}^{\prime } {\mathbf{x}}_{{\varvec{i}}}$ contains the *Q* × *S* matrix of coefficients **B** and the length-*Q* design vector **x**_*i*_. **Σ** is a *S* × *S* covariance matrix. There is a correlation matrix associated with Σ,

3 $${\mathbf{R}}_{{S,S^{\prime } }} = \frac{{{{\varvec{\Sigma}}}_{{S,S^{\prime } }} }}{{\sqrt {{{\varvec{\Sigma}}}_{S,S} {{\varvec{\Sigma}}}_{{S^{\prime } ,S^{\prime } }} } }}$$

The predictive distribution is obtained as

4 $$\left[\tilde{Y }\left| \tilde{X }\right.\right]=\int \left[ \tilde{Y }\left| \tilde{X }\right.,\widehat{\theta }\right]\left[\widehat{\theta } \left|X, Y\right.\right]$$

The integrand contains the likelihood (Eq. (2)) followed by the posterior distribution for parameters, $\widehat{\theta }= \left\{\widehat{\mathbf{B}},\boldsymbol{ }\widehat{{\varvec{\Sigma}}}\right\}$. Input $\tilde{X }$ can equal *X* (in-sample prediction) or not (out-of-sample prediction). We fitted both CPUE (continuous abundance) and presence-absence versions of the model. As a Bayesian method, GJAM provides probabilistic estimates of parameters with full dependence in data, including jointly distributed species. Model fitting is performed using Gibbs sampling, which is a Markov chain Monte Carlo (MCMC) technique.

The sensitivity of an individual response variable s to an individual predictor q is given by the coefficient βqs (individual coefficients from the B matrix). The sensitivity that applies to the full response matrix is given by

5 $${\mathbf{f}} = diag\left( {{\mathbf{B}}{\Sigma }^{ - 1} {\mathbf{B}}^{\prime } } \right)$$

The *Q* × *S* matrix **B** contains relationships of each species to the environment, the “signal”, but not to each another. Matrix **E** summarizes species similarities in terms of their response to an environment $\stackrel{\sim }{\mathbf{x}}$ and is given by

6 $${\mathbf{E}=\mathbf{B}}^{\boldsymbol{^{\prime}}}\mathbf{V}\mathbf{B}$$

where V is a covariance matrix for $\stackrel{\sim }{\mathbf{x}}$(a vector of predictors) and contributes the environmental component of variation in $\stackrel{\sim }{\mathbf{y}}$. Similar species in **E** have similar columns in **B**. Those similarities and differences are amplified for predictors $\stackrel{\sim }{\mathbf{x}}$ with large variance. Conversely, species differences in **B** do not matter for variables in **X** that do not vary. The covariance in predictors could come from observed data, i.e., the variance of **X** (see^12^ for more details).

Prior distributions for this study are non-informative. This is particularly helpful for the covariance, lending stability to Gibbs sampling and avoiding dominance by a prior. In cases this particular case, the direction of the prior effect may be known, but the magnitude is not.

#### Variable selection

Unlike the familiar univariate setting, variable selection has to consider which species are included in the model. In a univariate model, there is one response and perhaps a number of potential predictor variables from which to choose. As in a univariate model, variable selection focuses on predictors held in the *n* by *p* design matrix **X**. Rather than a response vector, the multivariate model includes the *n* by *S* response matrix **Y**. Unlike the univariate model, the overall fit and predictive capacity depends not only on what is in **X**, but also on the species that are included in **Y**, each of which would be best explained by a different combination of variables. Rare species having no signal will not provide cross-correlations and thus can offer little learning from an analysis. For this reason, there may be no reason to include them in model fitting. Given that many species may be rare, and rare types will not be explained by the model, there will be decisions about what variables to include on both sides of the likelihood (i.e., predictors and responses).

These considerations mean that simple rules for variable selection, such as the combination yielding the lowest DIC, may not be sensible. The combination of variables that yields the lowest DIC could miss variables that are important for subsets of species. In principle, one poorly-fitted species could dominate variable selection. The best model for responses ranging from rare to abundant will depend on precisely which species are included, both rare and abundant. Thus, in order to select variables, we utilize inverse prediction—predicting the environment from species – and the overall community sensitivity^12^.

**Figure 1:**
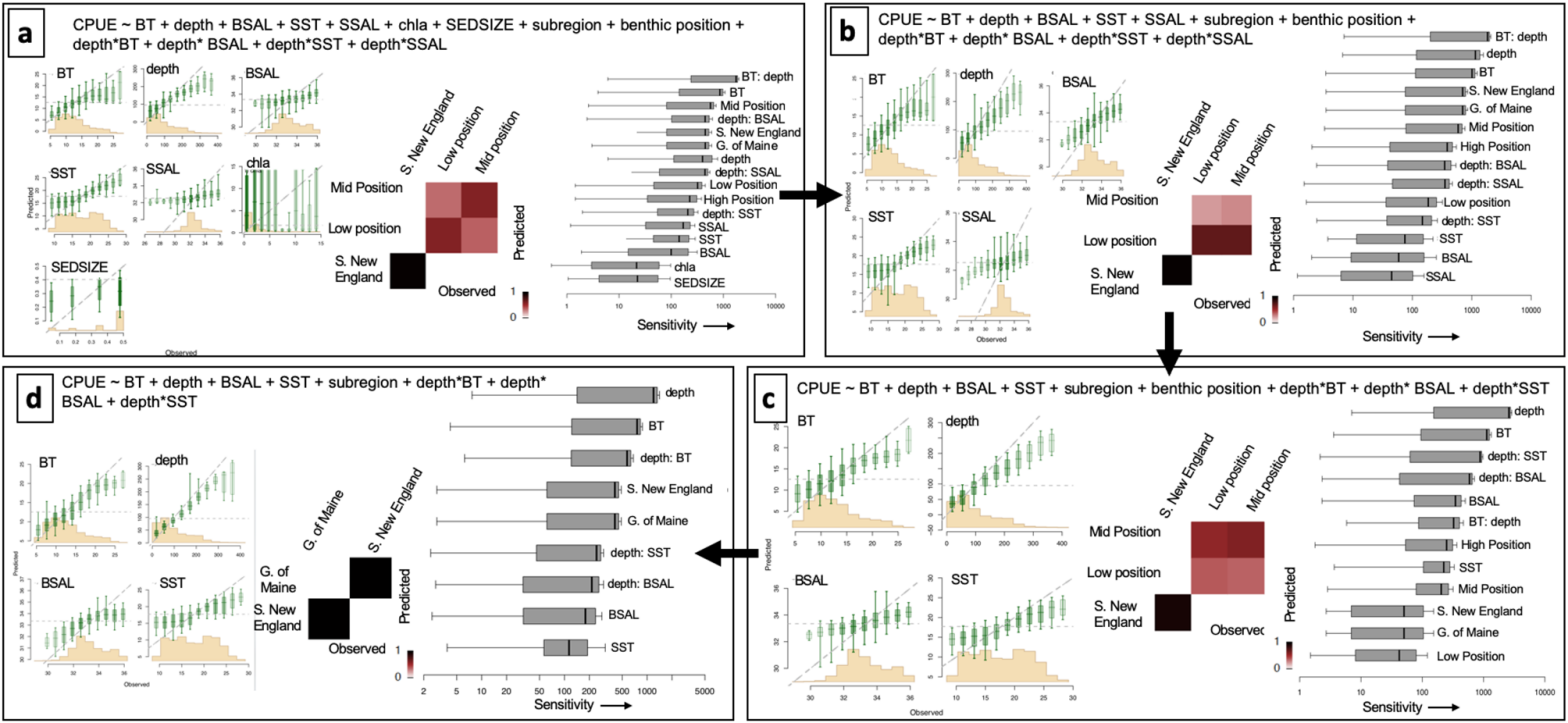
Inverse prediction and sensitivity for combinations of environmental parameters in GJAM. Starting with the most complicated model (**a**), sensitivity was highest for the interaction between BT and depth and lowest for Chla and sediment size (**a**). Inverse prediction confirms that sediment size and Chl_a_ contribute little to community biomass (**a**) and those are removed in the second model (**b**). SSAL contributes little to community response and are removed in the third model (**c**), The final model (**d**) includes terms that have strong inverse prediction and overall sensitivity. Inverse prediction for continuous and factor variables is on the left and center of each box, and overall sensitivity is on the right.

Inverse prediction provides a comprehensive estimate of the environmental importance for the entire community, because it determines the capacity of the community to predict (through the fitted model) the environment; it inverts the model^12^. A variable predicted by the community explains important variation in one to many species. A variable that is not predicted by the community does not explain important variation in any of them. To look at the importance of environmental variables for the entire community, we started with the saturated model that included the predictors BT, SST, depth, BSAL, SSAL, Chl_a_, SEDSIZE, subregion, benthic position and an interaction between depth and BT, BSAL, SST and SSAL (Fig. 1a). Sensitivity was highest for the interaction between BT and depth and lowest for Chla and sediment size (see right subpanel on Fig. 1a for sensitivity). Inverse prediction confirmed that sediment size and Chl_a_ contribute little to community biomass, because the community cannot “predict” them (see left and middle subpanels on Fig. 1a for sensitivity). Inverse prediction results from a second model (Fig. 1b) showed that SSAL and the third model for benthic position also (Fig. 1c) contribute little to the community response. Using the combination of sensitivity and inverse prediction we obtained the final model that includes BT, depth, BSAL, SST, subregion and an interaction between depth and BT, BSAL and SST (Fig. 1d). Inverse prediction indicates that the CPUE predicts the environment well. In the final model, sensitivity is highest for depth. Subregion remains as a two-level factor and there is strong inverse prediction for that variable as well (Fig. 1d). In the variable-selection stage, each model was run on the entire fall dataset for 5000 iterations and a burn-in of 800. Inverse prediction results from the spring model indicated similar patterns; thus, the same variables were used for the spring and fall.

We compare the model selected above using inverse prediction to a model selected using the more traditional method of out-of-sample prediction. For out-of-sample prediction, we fitted all combinations of 11 environmental variables (BT, BSAL, SST, SSAL, Chla, depth, sediment size, subregion, position, seabed form) plus interaction terms between depth and SEDSIZE, BT, BSAL, SST, SSAL and chlorophyll. These models were run with 1000 iterations and a burn-in of 400. All models included BT, BSAL, SST, SSAL, chlorophyll A and depth, as these variables have been shown to be important for these species. In total, 1,024 possible models were evaluated by training each potential model on 70% of the data (n = 3652 in the fall, n = 4155 in the spring), evaluating in-sample performance with DIC, and then testing out-of-sample performance on the remaining 30% (n = 1565 in the fall, n = 1780 in the spring). The 10 models with the lowest DIC in-sample were selected, and the final model was selected out of those 10 with the lowest out-of-sample R^2^. The selected model for fall CPUE had the following terms: ~ BT + depth + BSAL + SST + SSAL + chla + depth*BT + depth*SEDSIZE + depth*SSAL + depth*chla + SEDSIZE + Benthic position. Recall that inverse prediction selected a simpler model including the following terms: BT + depth + BSAL + SST + Subregion + depth*BT + depth*BSAL + depth*SST. The inclusion of SEDSIZE and benthic position in the model selected via out-of-sample prediction is probably a result of these predictor variables being important for a subset of species (i.e. benthic species^29^), but not the community as a whole. When we have a large number of response variables, as in this study, we need to consider the variables that are more important on a community level, rather than just for a few species. Thus, we use the model selected via inverse prediction for the remainder of the study.

We fitted the selected model with 70% of the data for 20,000 iterations with a burn-in of 8,000 iterations (n = 3652 in the fall, n = 4155 in the spring). Out-of-sample prediction was performed on the remaining 30% (n = 1565 in the fall, n = 1780 in the spring) of the dataset and predicted versus observed values were evaluated (Supplemental Figs. S2 and S3) as well as residual versus fitted values (Supplemental Figs. S4 and S5). As has been shown in other research^30,31^, aggregating noisy predictions based on similar environmental preferences can improve performance, especially for larger datasets. Thus, we generated an aggregated data set that uses a k-means clustering of predictors (Supplemental Figs. S8 and S9). We performed the same analysis for the spring and the fall as well as with the presence absence data and recorded AUC as well as precision for each species (Supplemental Figs. S6 and S7). Precision is defined as the arithmetic mean of precision (proportion of predicted presences actually observed as presences) across all threshold values (at an interval of 0.01).

#### Final model

We ran the final model on 100% of the data with 20,000 iterations and a burn-in of 8000 iterations for the spring and fall for CPUE as well as presence absence for a total of 4 models. From the final model we obtained coefficients for the species-environment responses, **β**, covariance between species in how they respond to the environment **E**, and the residual correlation from the fitted model, **R.** We subtracted the absolute values from the presence/absence residual correlation matrix from the absolute values of the CPUE residual correlation matrix to observe where these results diverged. For MCMC chains and convergence of the final model as well as example models from both methods of variable selection see Supplemental Figs. S10–S12).

#### Comparison to SSDMs

We built single species distribution models for each species in the form of GAMs using the mgcv package in R^32^. GAMs are a semiparametric extension of the generalized linear model and are a common modeling technique for species distribution modeling in this ecosystem^33^. For each species, we ran one GAM with CPUE as the response variable with a log-linked tweedie distribution that had penalized regression splines, a REML smoothing parameter with an outer Newton optimizer, 10 knots, and omitted NAs. We also ran GAMs for each species with a binary response variable indicating species presence with a binomial error distribution and a logit link function, penalized regression splines, a REML smoothing parameter with an outer Newton optimizer, 10 knots, and omitted NAs. We compared the out of sample observed versus predicted values for GAMs versus GJAM using RMSPE, R^2^, AUC, and precision. Root Mean Squared Prediction Error (RMSPE) is a measure of the average squared difference between the observed and predicted values, measured in the same units as the input data (kg/tow). R^2^ is a measure of the average squared difference between the observed and predicted values and is unitless. R^2^ is calculated as (1 − sum((predicted − observed)^2^)/sum((observed − mean(observed))^2^)) The ROC curve is a measure of model performance which plots true positive rate versus false positive rate, and the area under the ROC curve (AUC) provides a single measure of accuracy. A pairwise Wilcoxon test was used to compare means. We also compare the significance of predictors in both the GJAM model and GAM models. In this example, significance is defined for GJAM as a credible interval of the beta estimation that does not cross zero, and for the GAM as a p-value less than 0.05^34^.

#### Spatial and temporal autocorrelation

Examining the spatial and temporal autocorrelation of the modeled residuals can help specify missing endogenous (habitat selection or density dependence) and exogenous (covariate) effects that may be missing from the model. Thus, for each species modeled, we plot the spatial autocorrelation of residuals using a semi-variogram for the year 2015 and the temporal autocorrelation of the residuals using a partial autocorrelation function (PACF). We present the results for each species in the fall in the Supplemental materials (Supplemental Figs. S27–S57).

All analysis and figure creation was performed in R version 3.6.2^35^. Figures were created using the following R packages: ggplot2^36^, ggpubr^37^, corrplot^38^, gridExtra^39^, cowplot^40^, lessR^41^, and ggcorrplot^42^.

## Results

Both the univariate GAM and multivariate GJAM vary widely in their capacities to predict species abundances and presence. Out-of-sample prediction of each species from the CPUE GJAM models for the fall and spring is shown in Supplemental Figs. S2 and S3. We also show that clustering predictions and observations based on similar environmental gradients leads to less noisy and more interpretable observed versus predicted values and enhances performance for some species (Supplemental Figs. S8 and S9). This is likely due to unmodeled processes such as biological and sampling characteristics. Out of sample prediction for the GAM models is shown in Supplemental Figs. S25 and S26). Individual performance metrics can be found in Supplemental Table S1 and Supplemental Table S2. The mean R^2^ and Precision values differed significantly in the GAM model compared to the GJAM model in the spring and fall, with the GAM model performing better (Supplemental Fig. S23). The mean AUC and RMSE values did not differ between the GAM and GJAM models (Supplemental Fig. S24).

**Figure 2:**
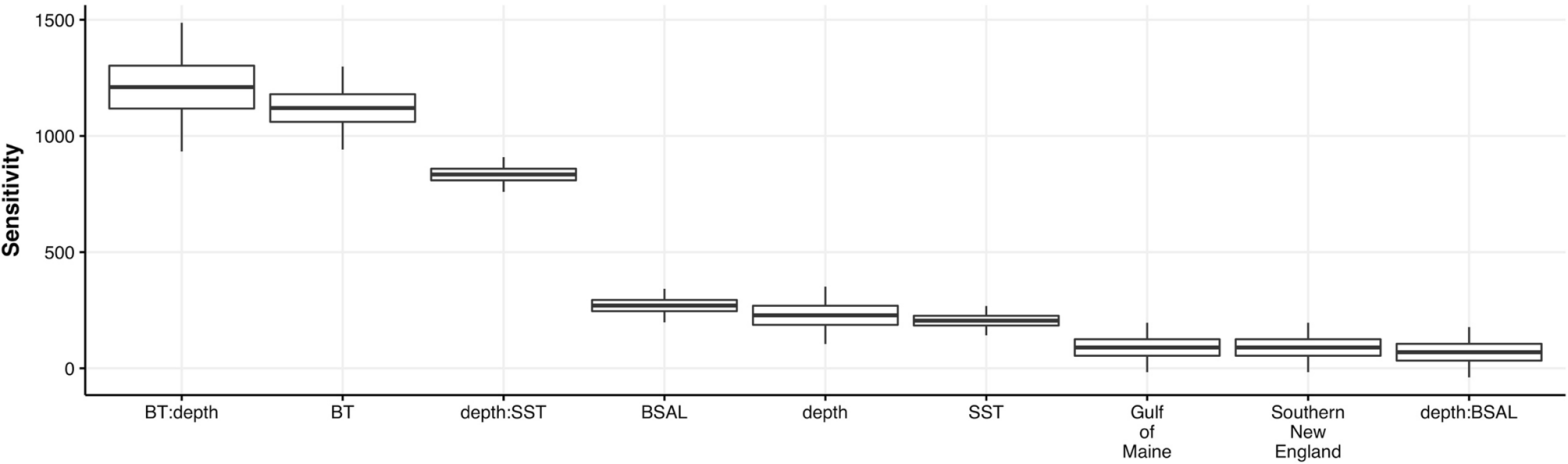
Environmental sensitivity for the entire community (CPUE) in the fall.

Unlike the GAM, by accounting for the dependence between species GJAM provides valid credible intervals on coefficients and, thus, allows inference on environmental effects. An interaction between depth and SST had the highest sensitivities across the entire community of species in the fall (Fig. 2) and subregion, BT, and depth had the highest sensitivities across the entire community of species in the spring (Supplemental Fig. S13). Our results from inverse prediction suggest that depth, BT and subregion influence the community (Fig. 1a) which agrees with the selected model sensitivity results (for environmental sensitivities from the presence/absence model, see Supplemental Figs. S14 and S15).

**Figure 3:**
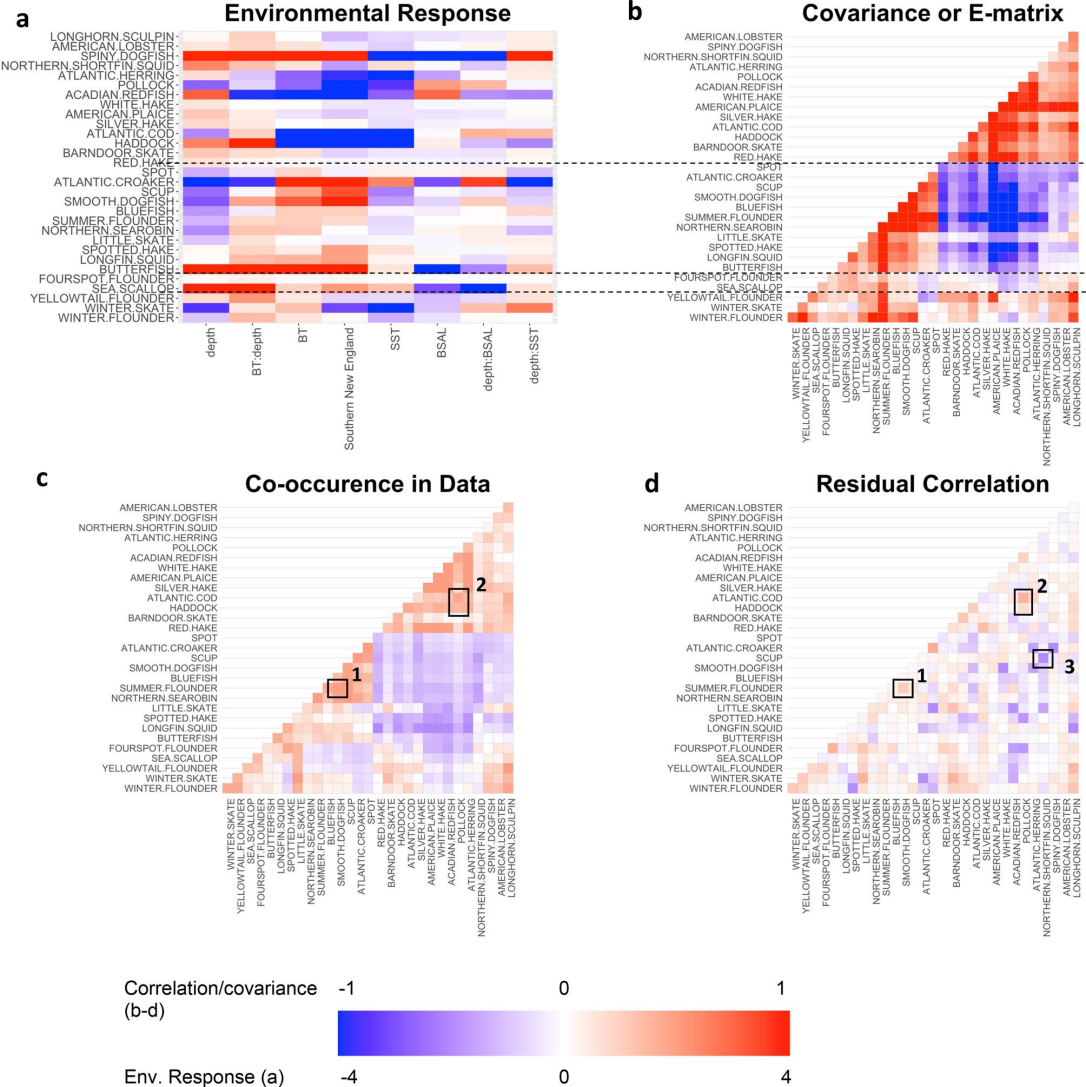
Model covariance results for the fall CPUE. (**a**) Coefficients for the species-environment responses (from fitted model), **β**. (**b**) Covariance between species in how they respond to the environment, **E**. (**c**) Species co-occurrence in catch data. (**d**) Residual correlation from the fitted model, **R**. Boxes highlight species discussed in the text. Environmental response of factor variable (**a**), subregion is compared to the baseline level (Gulf of Maine). Figure was created using the ggcorrplot (Version 0.1.3.999; http://www.sthda.com/english/wiki/ggcorrplot) and corplot (Version 0.84; https://github.com/taiyun/corrplot) packages in R.

Fitted coefficients from the GJAM model quantify the relationship of individual species’ CPUE to environmental conditions (Fig. 3a) and a measure of how species covary with environmental conditions (Fig. 3b). The similarities and differences between species that cause them to cluster in Fig. 3b come from environmental variables in design matrix **X**, both main effects and interactions. Species that covary with environmental variables (Fig. 3b) highlight two large groups, one group comprised of species that relate to deep, cool waters in the Gulf of Maine (Longhorn sculpin (*Myoxocephalus octodecemspinosus*), American lobster (*Homarus americanus*), spiny dogfish, northern shortfin squid (*Illex illecebrosus*), Atlantic herring (*Clupea harengus*), pollock, Acadian redfish, white hake, American plaice (*Hippoglossoides platessoides*), silver hake, Atlantic cod, haddock, barndoor skate and red hake(*Urophycis chuss*)). The other large group is comprised of shallow, warm water species found in Southern New England that primarily show a positive relationship with the interaction between bottom temperature and depth (spot (*Leiostomus xanthurus*), Atlantic croaker, scup (*Stenotomus chrysops*), smooth dogfish, bluefish, summer flounder, northern searobin (*Prionotus carolinus*), little skate (*Leucoraja erinacea*), spotted hake (*Urophycis regia*), longfin squid and butterfish (*Peprilus triacanthus*)). Sea scallop and fourspot flounder were grouped together as deep, warm water species that are primarily found in the less saline waters of Southern New England. Finally, winter skate (*Leucoraja ocellate*), winter flounder (*Pseudopleuronectes Americanus*), and yellowtail flounder (*Limanda ferruginea*) are grouped together and are related to shallow, warm bottom temperatures but cool surface temperature, less saline waters, and are found in the Gulf of Maine. Presence/absence clusters followed a similar pattern to clusters from the CPUE models (For spring CPUE results see Supplemental Fig. S16, for presence/absence results, see Supplemental Figs. S17 and S18, but, in general, the presence/absence model had larger residual correlation values than the CPUE model for most species, and there was more agreement between the models in the fall (Supplemental Fig. S19).

The residual correlation matrix could be utilized for conditional prediction, where the predicted abundance or probability of presence of one species can be inferred from the environment and the abundance or presence/absence of one (or more) other species. For example, our modeled results identify species that have been shown to co-occur in the literature (e.g., smooth dogfish and summer flounder or Atlantic cod, haddock and pollock), and in both our underlying data (boxes 1 and 2 Fig. 3c) and our modeled residual correlation (boxes 1 and 2 in Fig. 3d). This residual correlation between species in Fig. 3d provides the additional regression coefficients, whereby we could use information from one species (e.g., Atlantic cod or haddock) to aid in the prediction of another (e.g., pollock). For, these species have a high residual correlation value (box 2 in Fig. 3d). A large negative residual correlation between species, for example Northern shortfin squid and scup, could be the result of competition, a shared predator, or the many unmeasured variables (box 3 in Fig. 3d). The proportion of variance explained by the mean structure of the model can be found in Table 1. This provides insight on the magnitude of variance explained by the environmental covariates. When predicting species distributions, however, the spatial and temporal autocorrelation of the residuals should be considered. For, the spatial and temporal autocorrelation plots of the residuals demonstrate that most of these species are temporally and spatially correlated (Supplemental Figs. S27–S57).

**Table 1:** Total variance and Fraction of the total variance that comes from the mean.

Species	Total variance (fall)	Fraction explained by mean (fall)	Total variance (spring)	Fraction explained by mean (spring)
Alewife			0.354	0.0974
Atlantic herring	33.9	0.495	1.55	0.141
Barndoor skate	0.814	0.342		
Atlantic cod	34.9	0.539	36.8	0.381
Sea raven			0.368	0.367
American plaice	0.79	0.774		
American lobster	0.921	0.228	0.901	0.336
Northern shortfin squid	4.58	0.255		
Spot	3.54	0.369		
Little skate	1.21	0.292	6.65	0.372
Winter skate	20.2	0.414	5.35	0.128
Yellowtail flounder	2.12	0.465	1.69	0.5
Longfin squid	4.41	0.313	2.22	0.348
Haddock	332	0.408	418	0.366
Silver hake	0.784	0.349	0.355	0.211
Atlantic croaker	190	0.4		
Smooth dogfish	12.4	0.495		
Longhorn sculpin	1.2	0.555	1.5	0.476
Summer flounder	0.525	0.668	0.309	0.42
Fourspot flounder	0.0567	0.24	7.8	0.138
Butterfish	25.5	0.133	6.73	0.233
Sea scallop	8.63	0.315	2.39	0.155
Pollock	21.9	0.423	17.9	0.375
Bluefish	2.75	0.535		
Northern searobin	1.59	0.63	24.7	0.249
Winter flounder	1.88	0.47	1.08	0.51
Atlantic mackerel			51	0.042
Acadian redfish	135	0.546	197	0.66
Spiny dogfish	2440	0.251	1320	0.142
Scup	3.99	0.505		
Red hake	0.293	0.452	0.203	0.214
Spotted hake	1.45	0.416		
White hake	0.414	0.676	1.17	0.559

**Figure 4:**
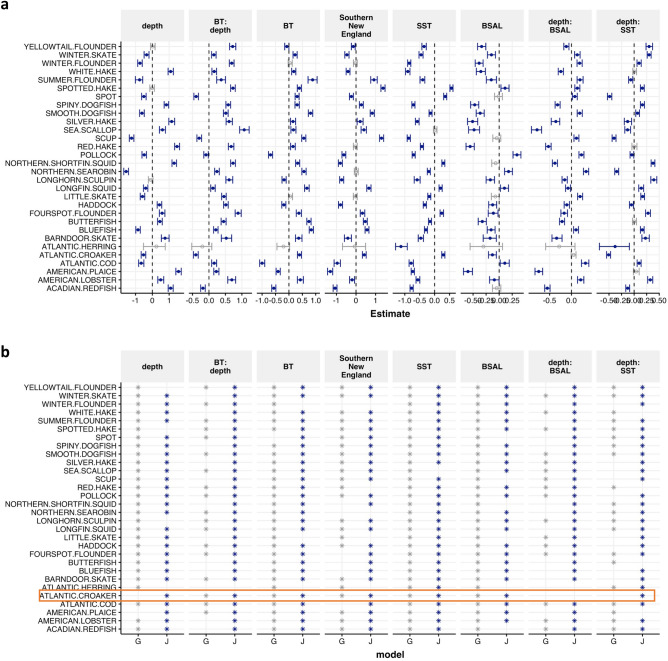
Beta sensitivities for individual species CPUE in the fall. Sensitivity of each species to beta parameters and estimated 95% credible intervals determined from GJAM model (**a**). Comparison of significant covariates in GAM (grey, G) versus GJAM (blue, J) models (**b**). Atlantic croaker is highlighted in orange.

Valid uncertainty estimates from GJAM allow us to evaluate significance of environmental effects in a probabilistic sense. Whereas credible intervals in Fig. 4a account for the dependence between species, the significance estimates for the GAM assume that species have been observed independently of one another (see Supplemental Fig. S20 for spring results and Supplemental Figs. S21 and S22 for presence/absence results). For example, depth is significantly related to Atlantic croaker biomass in GJAM (Fig. 4a), which is likely closer to ecological reality than the GAM model, which does not identify depth as important predictors for Atlantic croaker (Fig. 4b). Atlantic croaker is one of the rare species in this dataset (number of nonzero rows = 497 in the fall compared with the mean of 1655 nonzero rows for all species—Supplemental Table S1).

## Discussion

Models for the abundances of organisms within marine ecosystems must accommodate complex interactions and challenging data, including the mixture of continuous CPUE with (more commonly) discrete zeros. Despite a longtime awareness of the need for ecosystem-based fisheries management that incorporates species dependencies^4,43^ and a global and regional push through international agreements and regional planning actions^43^, progress towards ecosystem-based fisheries management in practice remains scarce^6^. Our results demonstrated that JSDMs and GAMs performed similarly in terms of out-of-sample prediction metrics such as AUC and RMSPE; however, the joint modeling approach provides accurate estimates of uncertainty that accounts for co-dependence and proper treatment of zeros. The GAM model generates significance tests that assumes independent observation of each species, which is hard to square with the knowledge that all of the species come from the same trawls. Jointly modeling species allows us to identify how species respond to the environment while accounting for the co-dependence between species and potential dependence between species inferred from the correlation matrix, which can be used for conditional prediction. We discuss each of these advantages below.

By selecting variables through inverse prediction, we are able to identify which variables are influencing the community as a whole while also preventing one or a few poorly-fitted species to dominate variable selection. The selected variables have been shown to influence species distributions in this area^14,15,29^ either through influences on species metabolic demand (temperature), benthic habitat preferences (depth), or prey availability as a result of stratification (temperature, salinity). The importance of temperature and depth has been documented in other studies^15^. The importance of interactions between depth and salinity may be explained by: (1) the Gulf Stream—offshore relatively deep, warm waters, with upwelling that stimulates productivity in the southern region of this study^45^; (2) the Labrador Current—inshore cool, fresh water from the Labrador shelf in the northern portion of this study^46^; and/or (3) seasonal offshore migrations that have been documents for several of these species^47^. The inclusion of sediment size and benthic position when selecting variables via DIC versus inverse prediction, which removes these variables, is likely the result of these variables being important for several species, but not the community as a whole. Indeed, several benthic species’ distributions in this dataset are more likely driven by benthic habitat characteristics^29^, while the community as a whole is more driven by temperature and depth. The strong influence of bottom salinity in the spring is perhaps due to high precipitation and freshwater runoff in the area^48^. The covariance matrix is supported by literature; Atlantic cod, haddock, and pollock covary with the environment and also co-occur in the underlying dataset (Fig. 2b,c) and are a commonly documented species assemblage^14,49^.

The hierarchical structure of this particular JSDM may allow for sharing of information between functionally similar species, which may be especially useful for rare species (i.e. Atlantic croaker in this study)^50^. Two functionally similar species, such as Atlantic croaker and scup, which respond similarly to the environment can jointly use information and inform prediction. Atlantic croaker may especially benefit from sharing information from more common species, as this commercially important species may redistribute under continued ocean warming as they are a more southern species.

When inference is done on multiple species, the joint model provides valid credible intervals needed to assign probability to environmental effects. Modeling the influence of temperature on each species independently can lead to conclusions that too many or too few species show significant responses. Warton et al.^51^ showed that ignoring correlation across species (i.e. not including latent variables in the model) results in 95% credible intervals that were too narrow and did not capture the observed value for species richness sufficiently; however, latent variable models, like the one presented here, which accounted for correlation across species, had wider interval widths, closer to 95% coverage of observed richness^51^. Our results demonstrate that incorporating correlation across species may result in more accurate environmental responses across species.

The combination of estimates of environmental effects and the environmental covariance from this joint model can be used to infer species similarities and ultimately aid in understanding the effects of changing ocean conditions on entire species assemblages. Recent work has attempted to identify historical shifts in ecological assemblages in this area using clusters based on environmental associations^52^. Future work could incorporate the findings from our study, which can identify assemblages based on environmental responses as well as environmental covariances. This environmental covariance can account for the fact that species that respond in similar directions to certain environmental variables and covary strongly with the same environmental variable may respond differently to changing ocean conditions than species that respond in similar directions but do not covary as strongly.

The residual correlation in this JSDM gathers model misspecification, missing environmental covariates, and biotic dependencies into pairwise coefficients for unexplained variation, and can ultimately be exploited for conditional prediction^50^. For instance, one species (such as scup) can be used to predict the probability of presence or abundance of another species (such as Atlantic croaker). While SSDMs can conditionally predict species based on the product of marginal occurrence probabilities, this method fails to account for interspecific correlations^50^. With JSDMs, the probability of co-occurrence also depends on the residual correlations with positively correlated residuals leading to higher probabilities of co-occurrence than SSDMs^50^. The ability of JSDMs to account for residual correlations in conditional prediction will be especially important when predicting species abundances along a variety of environmental gradients. For instance, biotic dependencies will likely play a critical role in the restructuring of communities under climate change and the ability of JSDMs to potentially account for dependence between species remains especially important when predicting future distributions under a changing climate. Given the temporal and spatial autocorrelation of the residuals, a critical next step will be to incorporate these correlations in a joint modeling framework. A time series version of GJAM has been applied to terrestrial settings^53^ and the spatiotemporal VAST model has been applied to other marine systems^54^. Additionally, spatiotemporal models at the individual species level^55^ have been developed. Thus, combining these efforts to create a joint spatiotemporal model of the NEUS ecosystem will allow managers to estimate species distributions conditioned on both the presence of other species, the environment, and spatial covariance.

## Conclusion

We provide one example of a joint species distribution model of the Northwest Atlantic using a Bayesian hierarchical modeling framework. In general, the added information gleaned from joint modeling will prove especially useful when predicting species distributions in a changing climate as well as current distributions for rare species. Inferring future distributions based not only on environmental conditions but also on the co-occurrence of other species will help generate more biologically robust forecasts. Leveraging conditional predictions based on the residual correlations found through a joint modeling framework like the one presented here is a clear and simple step towards achieving this goal. For, models that consider joint distributions and spatial and temporal components would be especially useful considering the imminent marine spatial planning changes in the area. For instance, offshore wind will require models that not only determine a current species' distribution, but also account for species movement through space and time. Similarly, the effects of offshore wind on one species will mostly likely depend on the environment as well as other species in the area, requiring a hierarchical joint modeling framework.

## Supplementary Information

Supplementary Information.

Supplementary Figures.

## Data Availability

All of the data analyzed in this study are publicly available. NEFSC bottom trawl data may be downloaded from OceanAdapt (https://oceanadapt.rutgers.edu). The benthic habitat data can be downloaded from http://www.conservationgateway.org.
