# Supplementary material for: Jointly modeling marine species to inform the effects of environmental change on an ecological community in the Northwest Atlantic
Source: Sci Rep. 2022 Jan 7;12:132. doi: 10.1038/s41598-021-04110-0 (PMC8742080; doi:10.1038/s41598-021-04110-0)
Supplement: Supplementary file 1 — Supplementary Information. [file 41598_2021_4110_MOESM1_ESM.docx]

Supplemental Figures


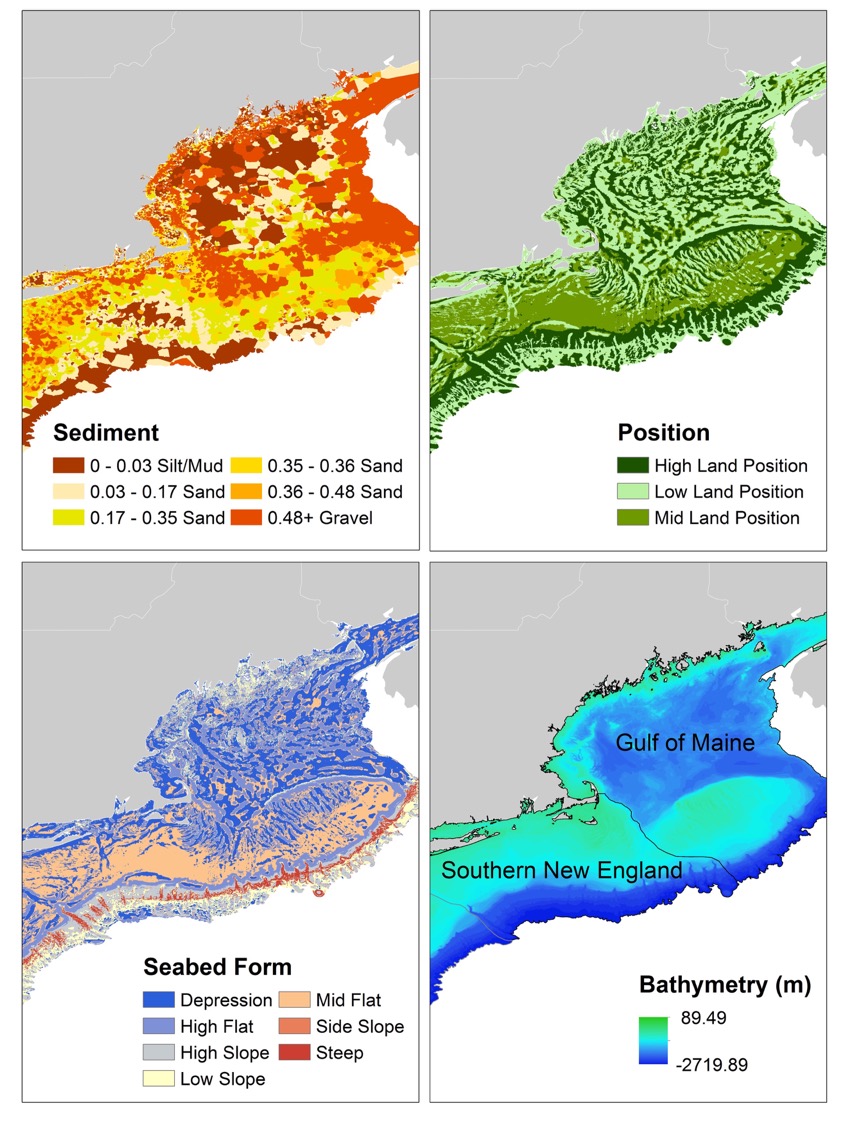


**Supplemental Figure S1.** Sediment size, benthic position, seabed form, and bathymetry data used for analysis. Subregion is denoted on top of bathymetry raster. Seabed form and benthic position were related, so we used benthic position to refrain from using a large number of factor variables. Figure was created using ArcMap Version 10.8 (<https://support.esri.com/en/products/desktop/arcgis-desktop/arcmap/10-8>)


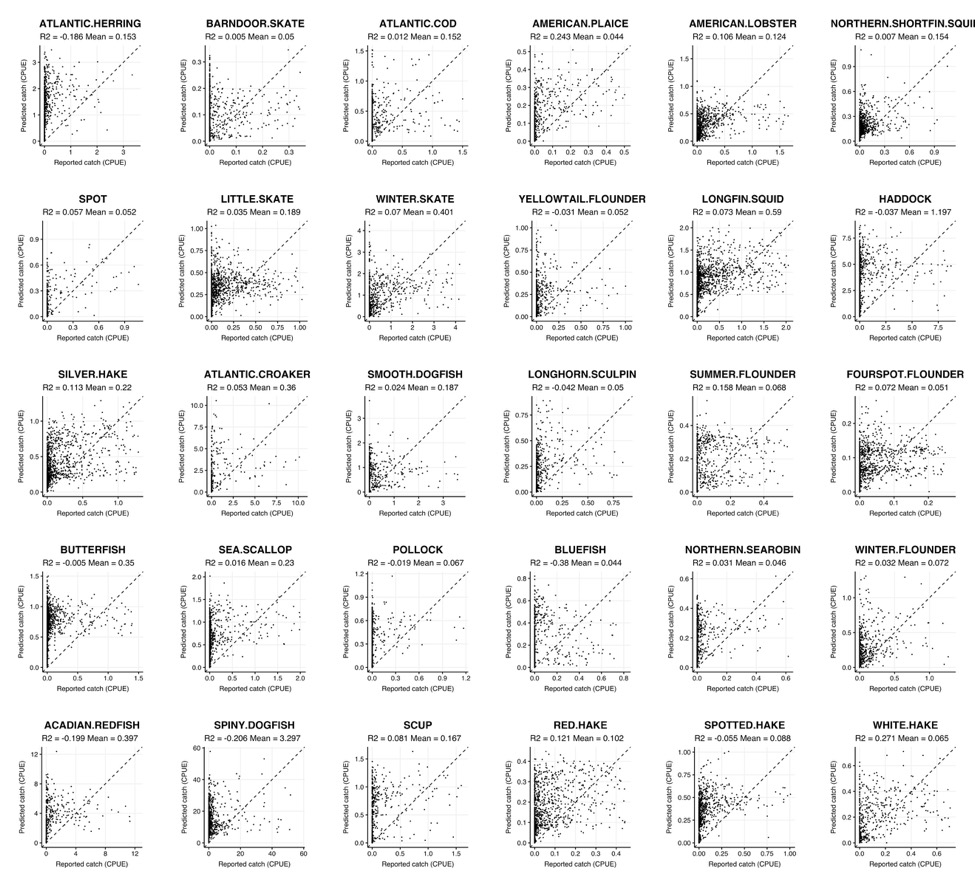


**Supplemental Figure S2. Out of sample prediction for fall species CPUE using GJAM.** RMSE and mean CPUE values noted in subtitle.


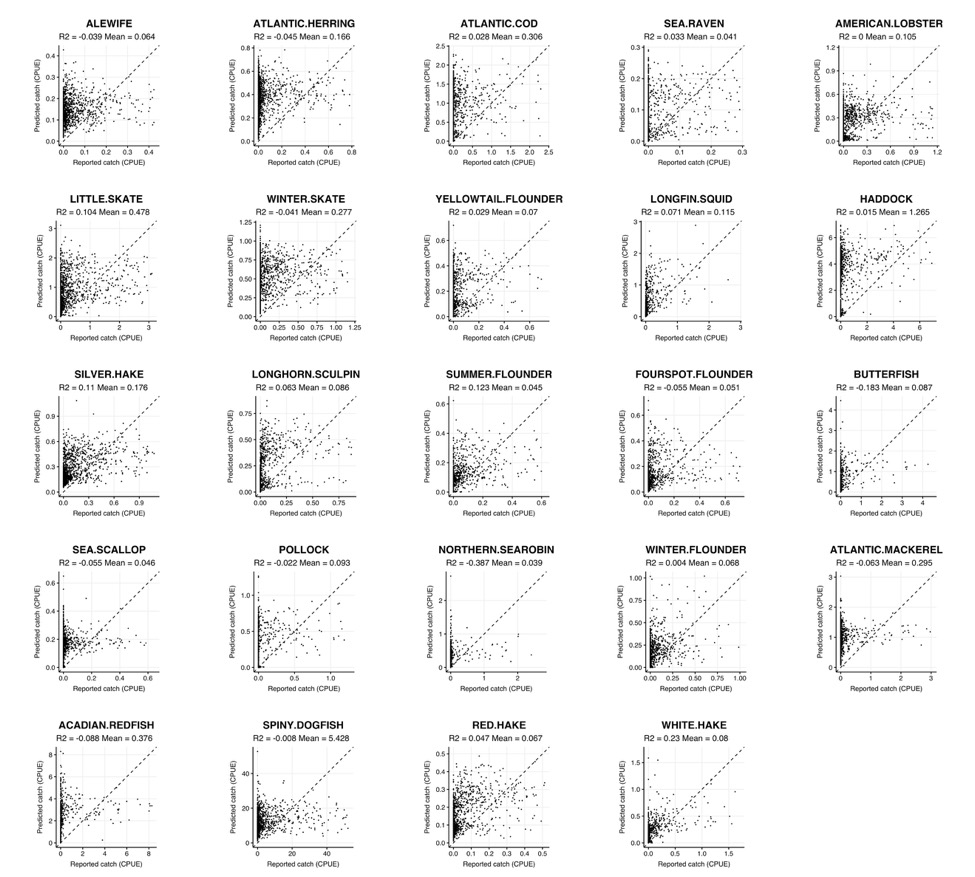


**Supplemental Figure S3. Out of sample prediction for spring species CPUE using GJAM.** RMSE and mean CPUE values noted in subtitle.


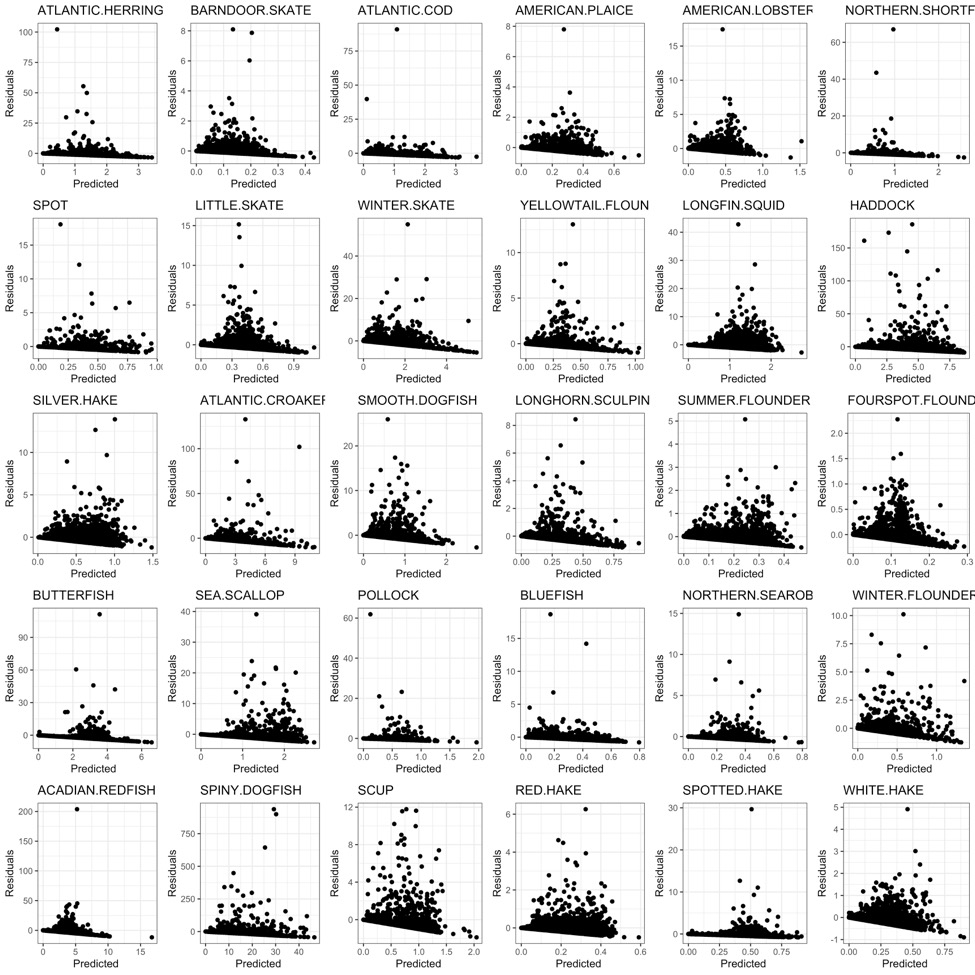


**Supplemental Figure S4. Residuals vs. fitted values for the fall CPUE GJAM model.**

**
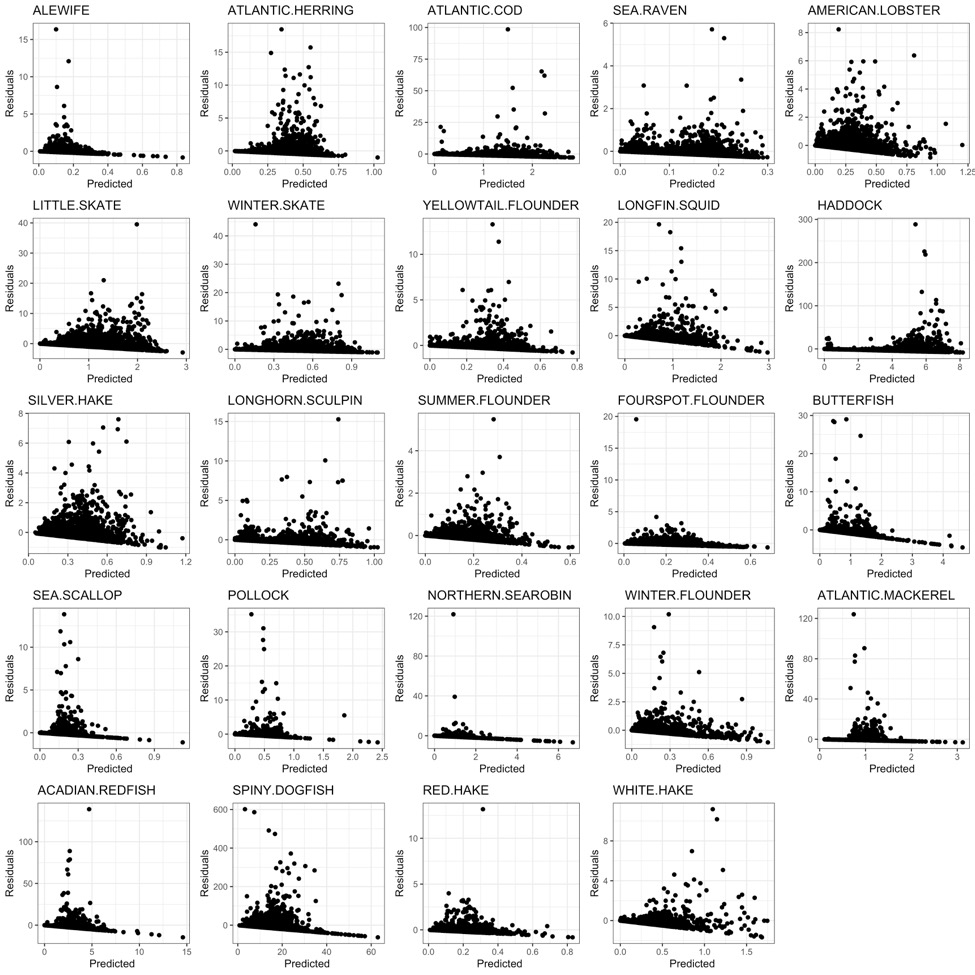
**

**Supplemental Figure S5. Residuals vs. fitted values for the spring CPUE GJAM model.**


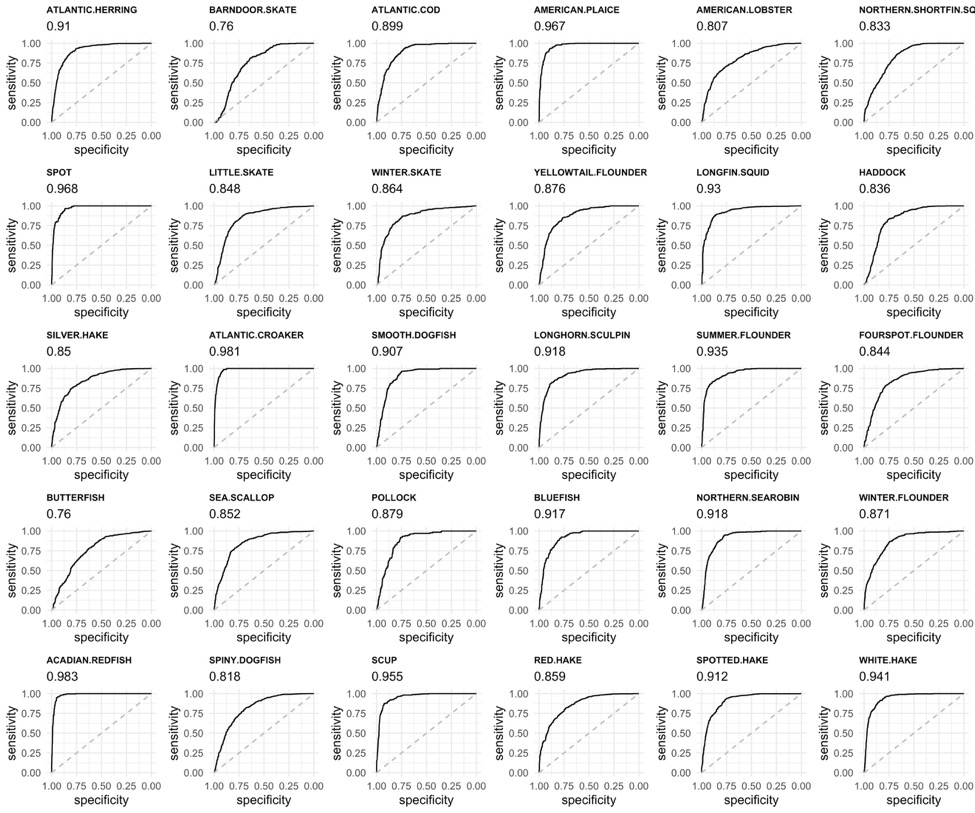


**Supplemental Figure S6. Receiver operator curve for out of sample prediction for fall species presence using GJAM.** Area under the ROC curve values noted in subtitle.


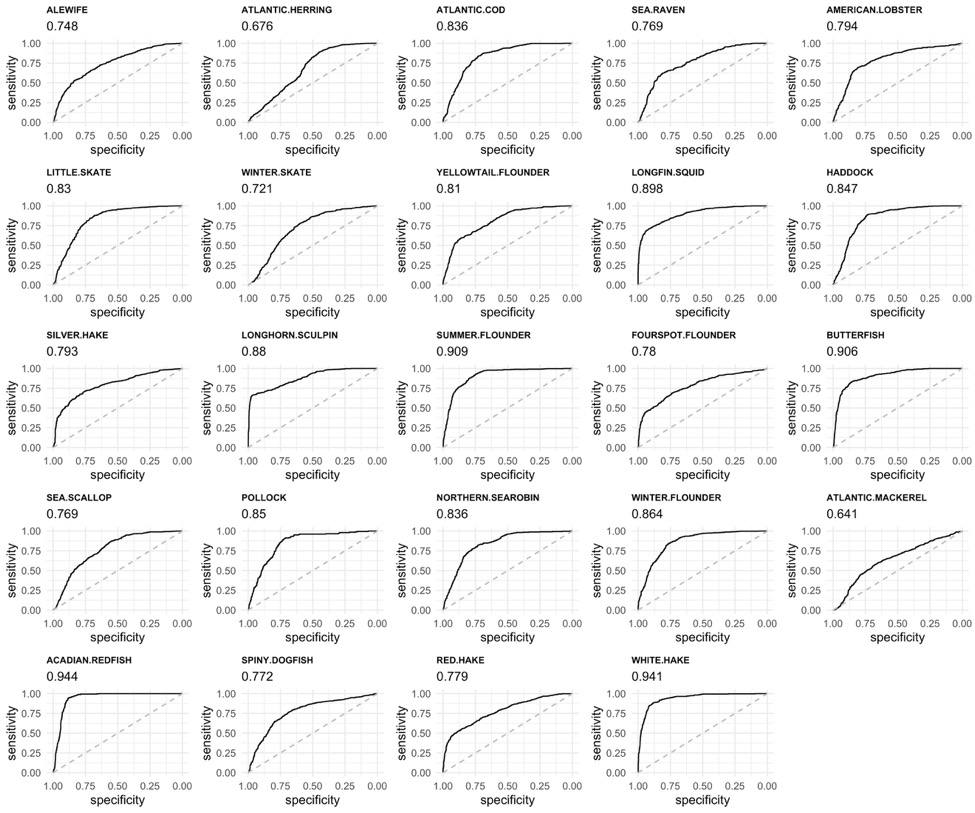


**Supplemental Figure S7. Receiver operator curve for out of sample prediction for spring species presence using GJAM.** Area under the ROC curve values noted in subtitle.


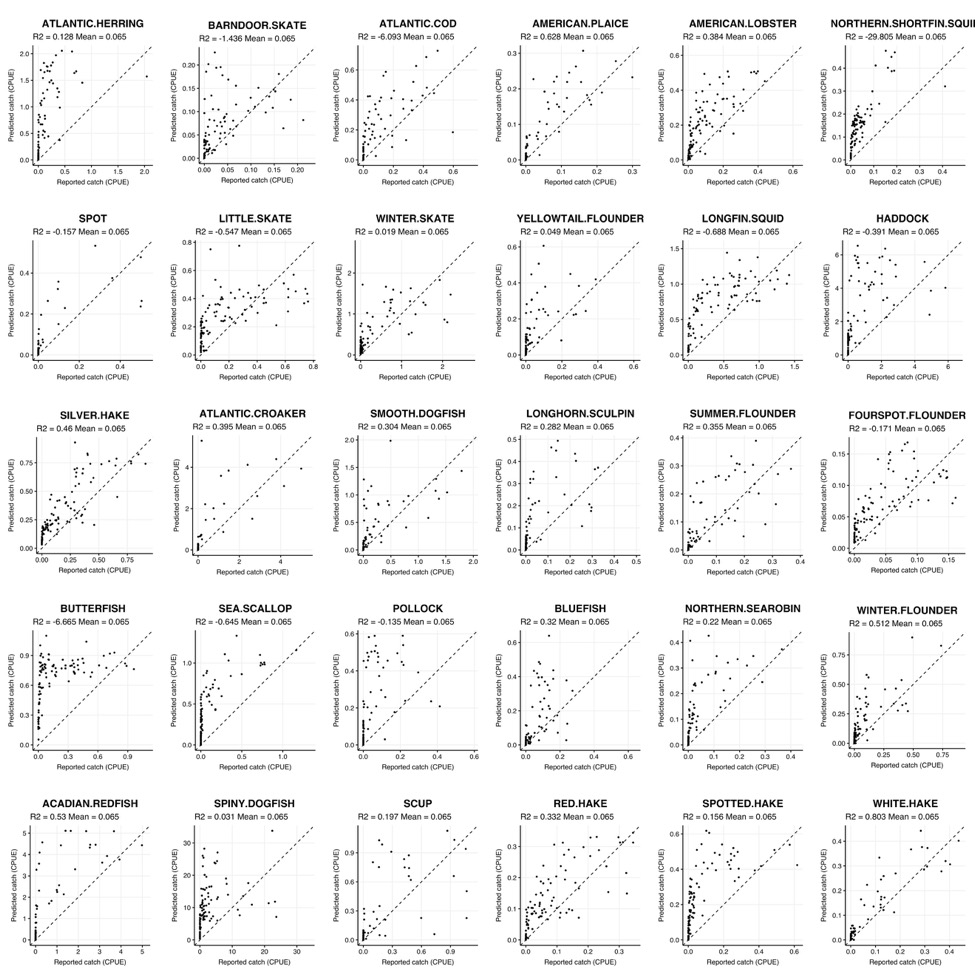


**Supplemental Figure S8. Out of sample prediction for fall species CPUE using GJAM.** Points are clustered based on similar environmental variables (using K-means clustering)


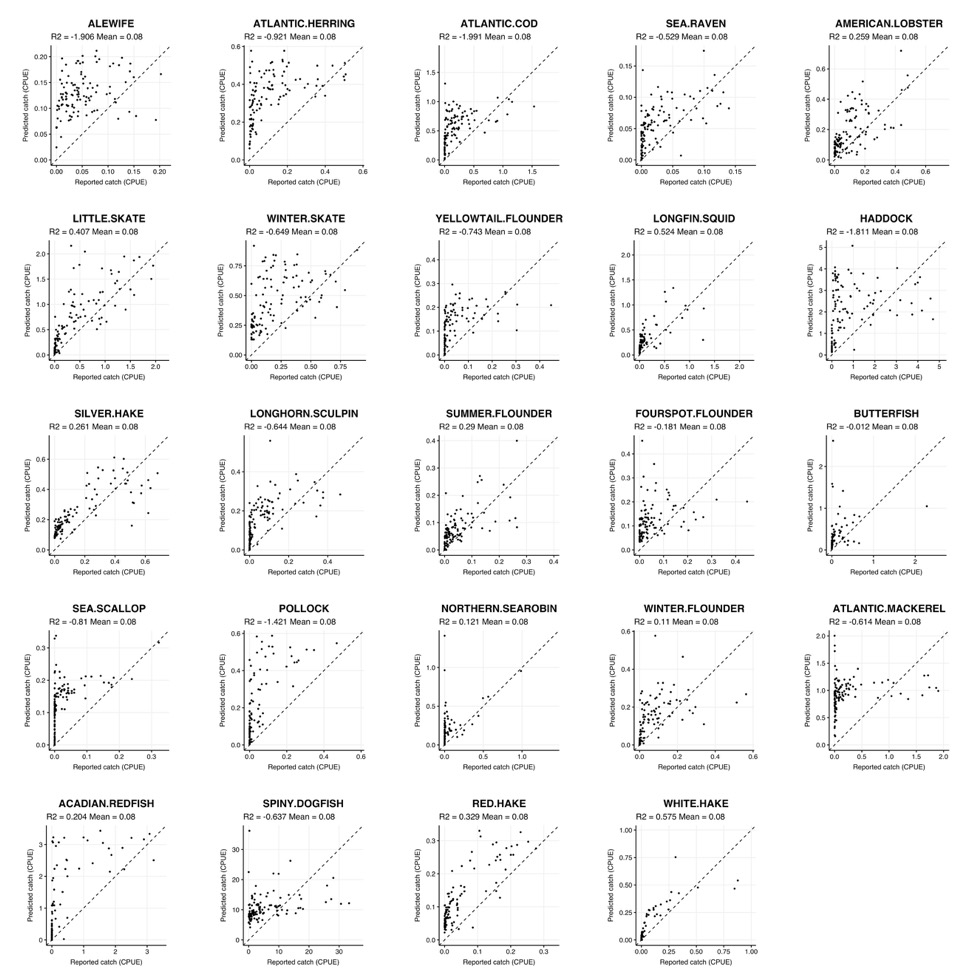


**Supplemental Figure S9. Out of sample prediction for spring species CPUE using GJAM.** Points are clustered based on similar environmental variables (using K-means clustering)

**Supplemental Figure S10. MCMC chains for final model (betas) in the fall.** Model was run with 20,000 iterations and a burnin of 8,000.

**Supplemental Figure S11. MCMC chains for model used (betas) for variable selection in the fall.** Model was run with 50,000 iterations and a burnin of 800.

**Supplemental Figure S12. MCMC chains for final model (betas) in the fall.** Model was run with 20,000 iterations and a burnin of 8,000.


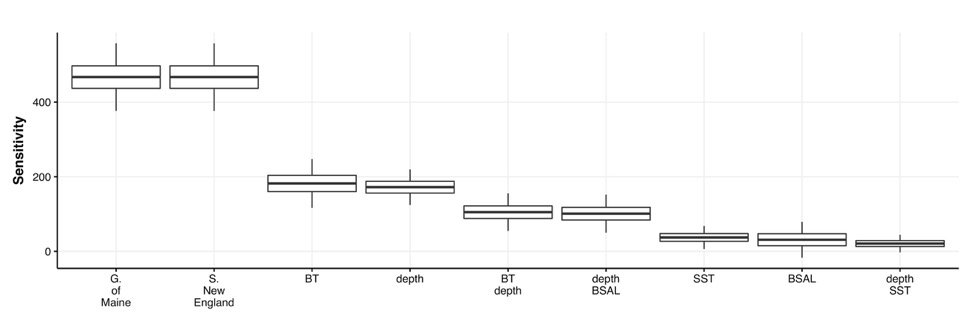


**Supplemental Figure S13.** Environmental sensitivity for the entire community (CPUE) in the spring.


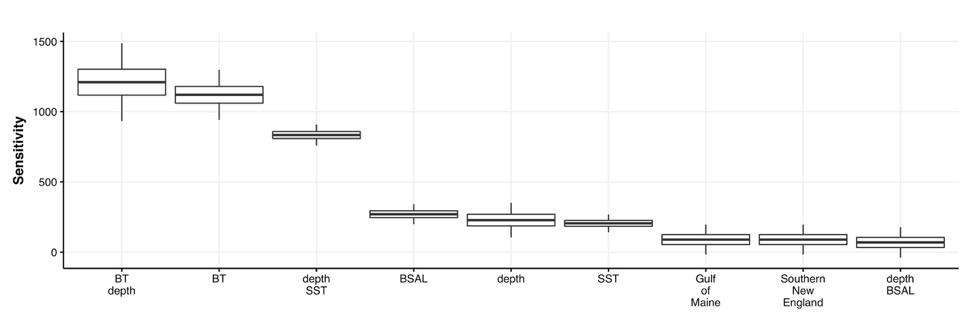


**Supplemental Figure S14.** Environmental sensitivity for the entire community (presence) in the fall.


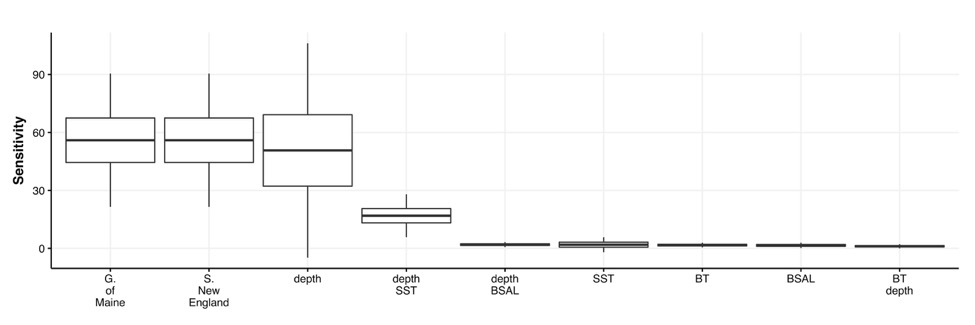


**Supplemental Figure S15.** Environmental sensitivity for the entire community (presence) in the fall.

In the spring, species that covary with environmental variables highlight three large groups. One group comprised of species that relate to warm waters in Southern New England and are primarily associated a negative interaction between depth and bottom salinity (Spiny dogfish, fourspot flounder, longfin squid, butterfish, atlantic mackerel, summer flounder and northern searobin). The second group is comprised of species that are related to deep, cool waters in the Gulf of Maine (silver hake, red hake, white hake, alewife, atlantic cod, pollock, acadian redfish, american lobster and haddock). Sea raven, longhorn sculpin, atlantic herring, yellowtail flounder and winter flounder are grouped together and are associated with shallow, fresh waters in the gulf of Maine. Finally, sea scallop, little skate and winter skate group together. We are also able to identify species that co-occur in the underlying data (box in Supplemental Figure S11c) and our modeled residual correlation (box in Figure S11d) – summer flounder and butterfish.


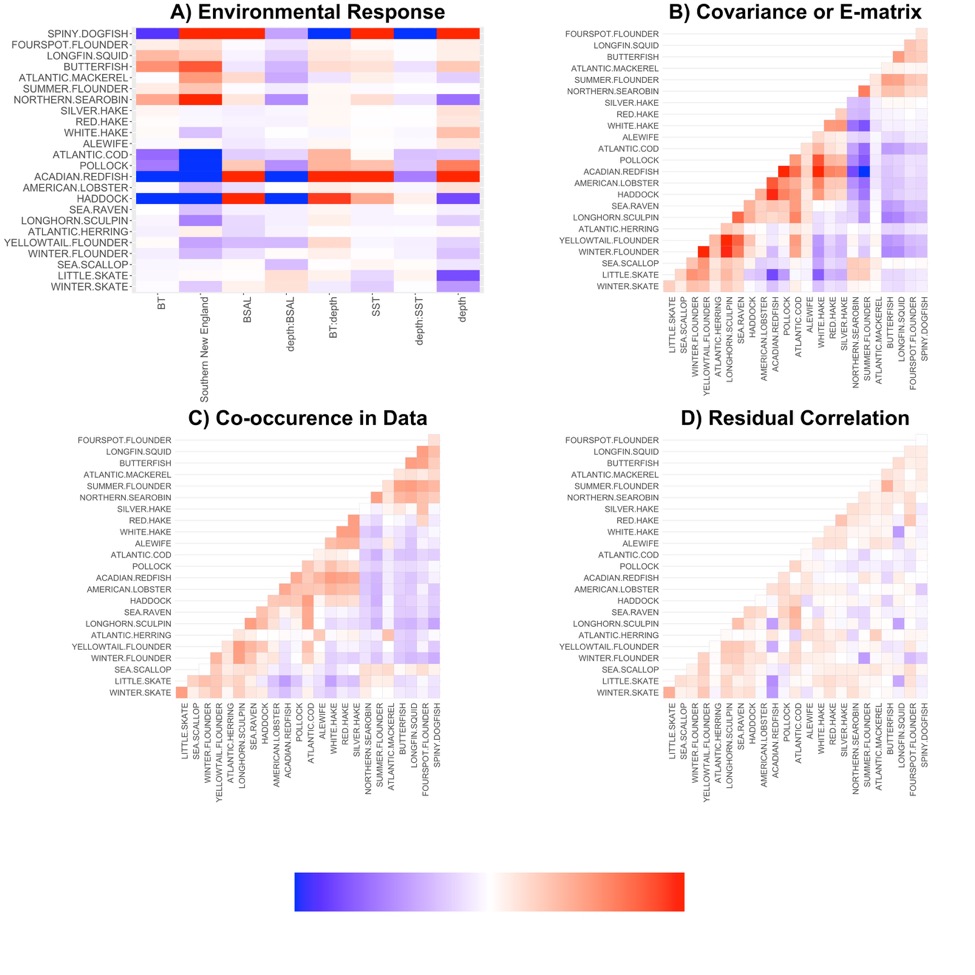


Correlation/covariance -1 0 1

(B-D)

Env. Response (A) -4 0 4

**Supplemental Figure S16. Model covariance results for the spring CPUE.**  (A) Coefficients for the species-environment responses (from fitted model), **β**. (B) Covariance between species in how they respond to the environment, **E**. (C) Species co-occurrence in catch data. (D) Residual correlation from the fitted model, **R**. Environmental response of factor variables (a), subregion are compared to the baseline level (Gulf of Maine). Figure was created using the ggcorrplot (Version 0.1.3.999; http://www.sthda.com/english/wiki/ggcorrplot) and corplot (Version 0.84; https://github.com/taiyun/corrplot) packages in R.

**
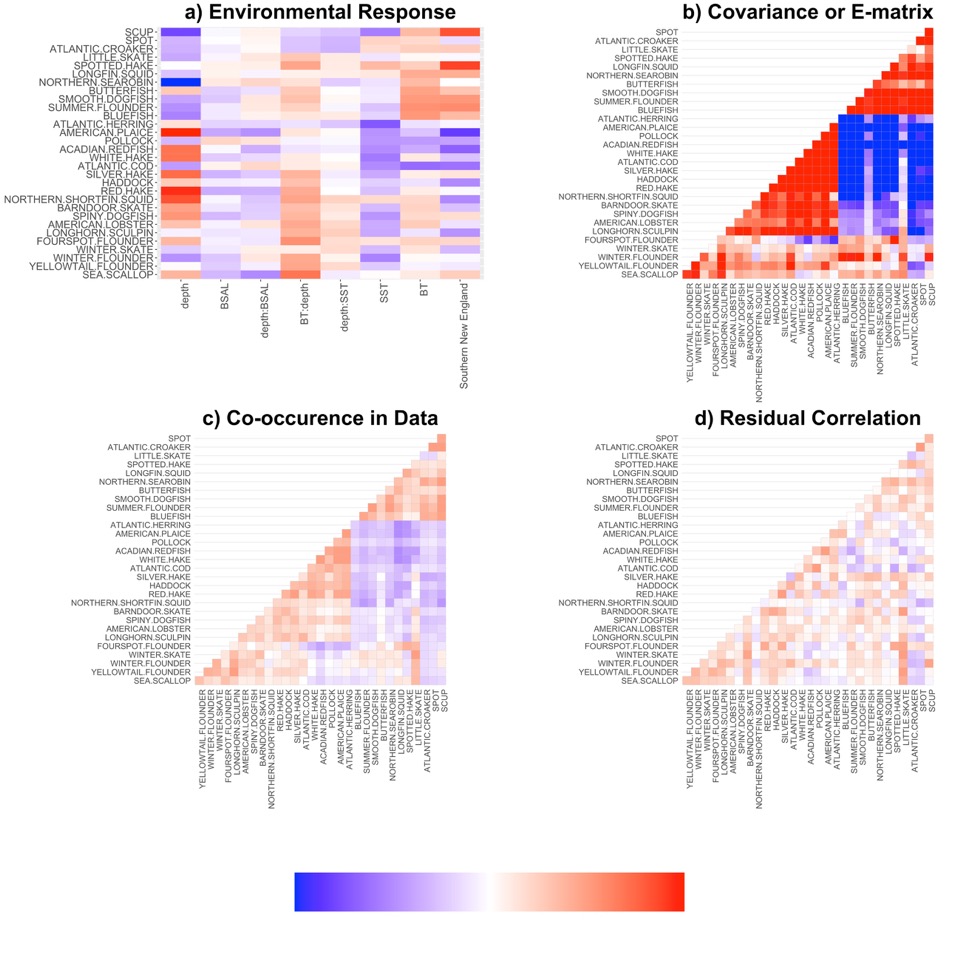
Supplemental Figure S17. Model covariance results for the fall presence.**  (A) Coefficients for the species-environment responses (from fitted model), **β**. (B) Covariance between species in how they respond to the environment, **E**. (C) Species co-occurrence in catch data. (D) Residual correlation from the fitted model, **R**. Environmental response of factor variables (a), subregion are compared to the baseline level (Gulf of Maine). Figure was created using the ggcorrplot (Version 0.1.3.999; http://www.sthda.com/english/wiki/ggcorrplot) and corplot (Version 0.84; https://github.com/taiyun/corrplot) packages in R.

Correlation/covariance -1 0 1

(B-D)

Env. Response (A) -4 0 4


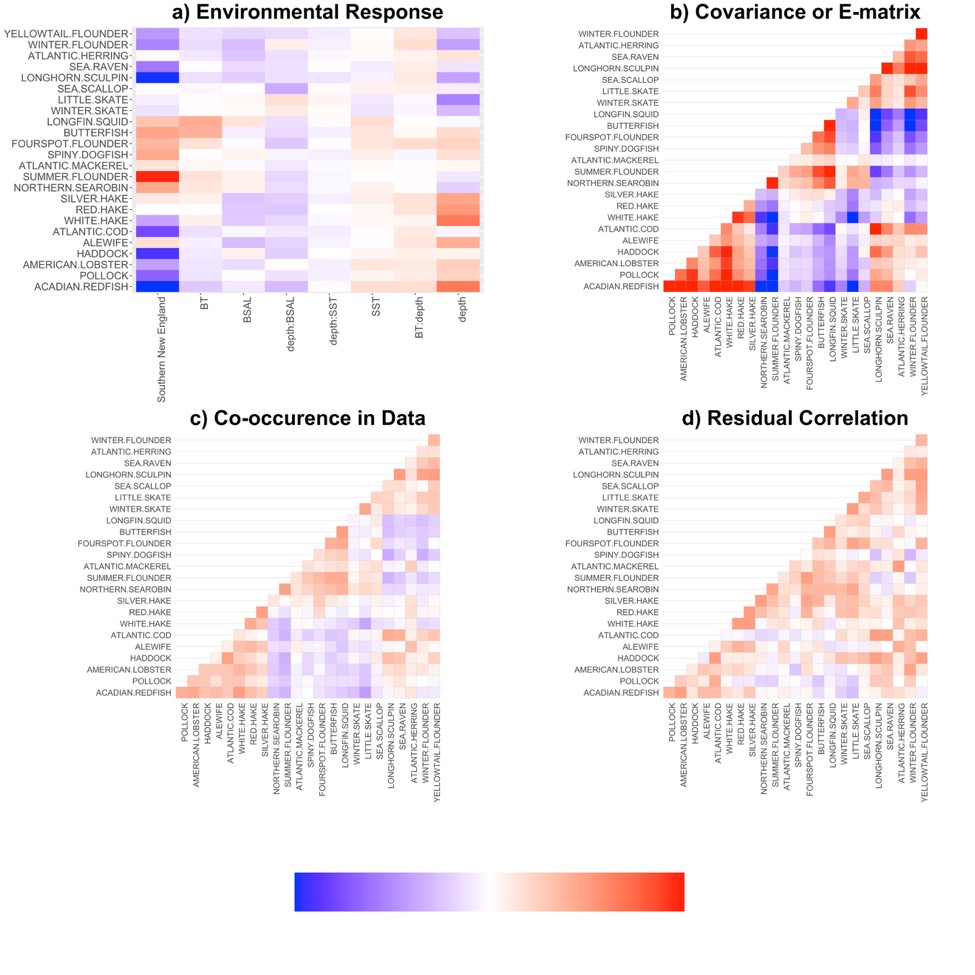


Correlation/covariance -1 0 1

(B-D)

Env. Response (A) -4 0 4

**Supplemental Figure S18. Model covariance results for the spring presence.**  (A) Coefficients for the species-environment responses (from fitted model), **β**. (B) Covariance between species in how they respond to the environment, **E**. (C) Species co-occurrence in catch data. (D) Residual correlation from the fitted model, **R**. Environmental response of factor variables (a), subregion are compared to the baseline level (Gulf of Maine). Figure was created using the ggcorrplot (Version 0.1.3.999; http://www.sthda.com/english/wiki/ggcorrplot) and corplot (Version 0.84; https://github.com/taiyun/corrplot) packages in R.


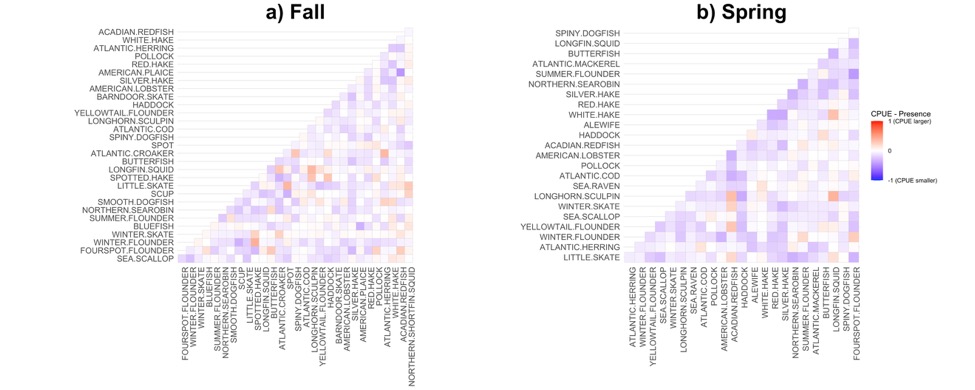


**Supplemental Figure S19. Residual correlation matrix agreement between CPUE and presence/absence model.** Absolute value of the CPUE residual correlation matrix minus the absolute value of the Presence residual correlation matrix for the fall (a) and spring(b). White values represent species residual correlation that was similar across models, red values represent residual correlation values that were larger in the CPUE model, and blue values represent residual correlation values that were larger in the presence/absence model. Figure was created using the ggcorrplot (Version 0.1.3.999; http://www.sthda.com/english/wiki/ggcorrplot) and corplot (Version 0.84; https://github.com/taiyun/corrplot) packages in R.


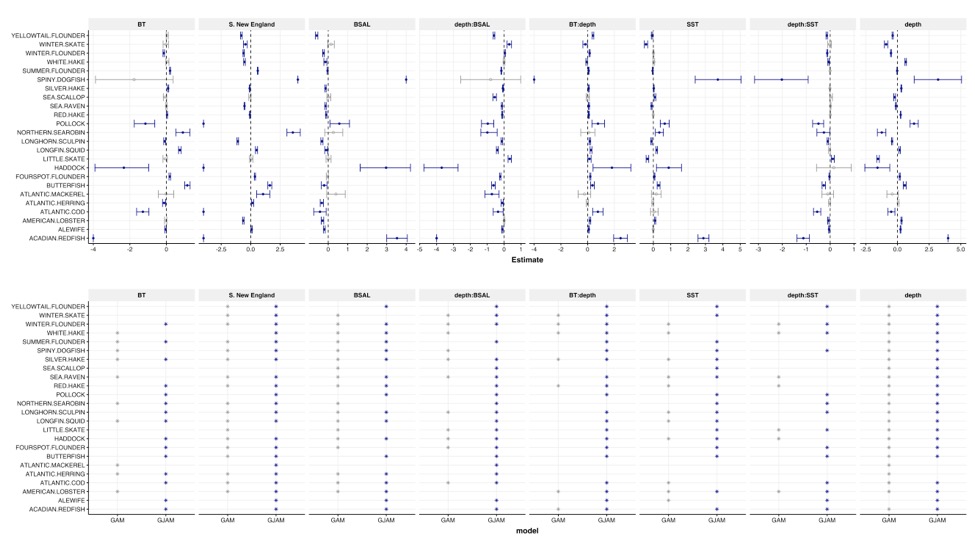


**Supplemental Figure S20. Beta sensitivities for individual species CPUE in the Spring.** Sensitivity of each species to beta parameters and estimated 95% credible intervals determined from GJAM model (a). Comparison of significant covariates in GAM (grey) vs. GJAM (blue) models (b).


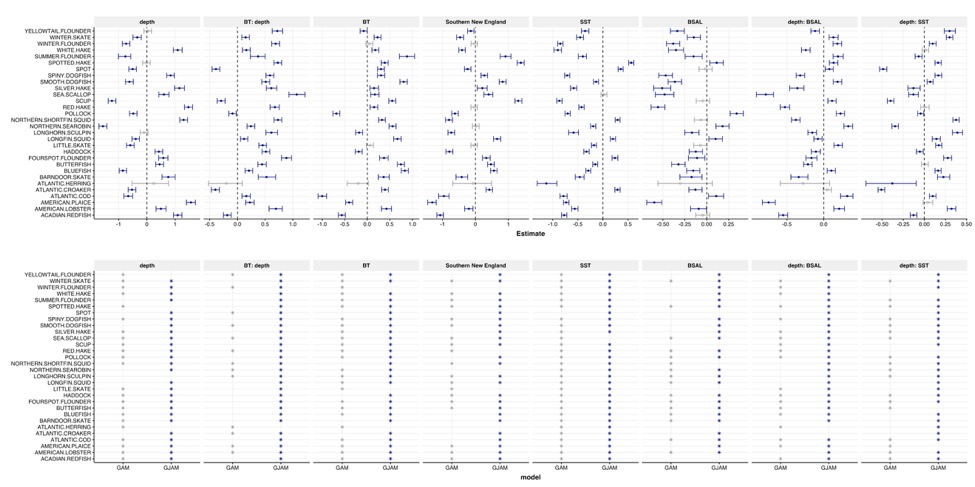


**Supplemental Figure S21. Beta sensitivities for individual presence in the fall.** Sensitivity of each species to beta parameters and estimated 95% credible intervals determined from GJAM model (a). Comparison of significant covariates in GAM (grey) vs. GJAM (blue) models (b).


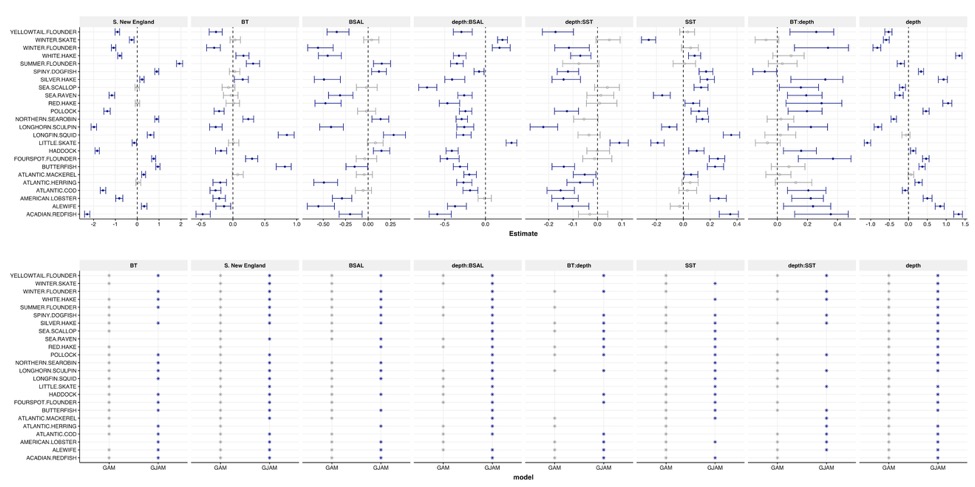
**Supplemental Figure S22. Beta sensitivities for individual presence in the spring.** Sensitivity of each species to beta parameters and estimated 95% credible intervals determined from GJAM model (a). Comparison of significant covariates in GAM (grey) vs. GJAM (blue) models (b).


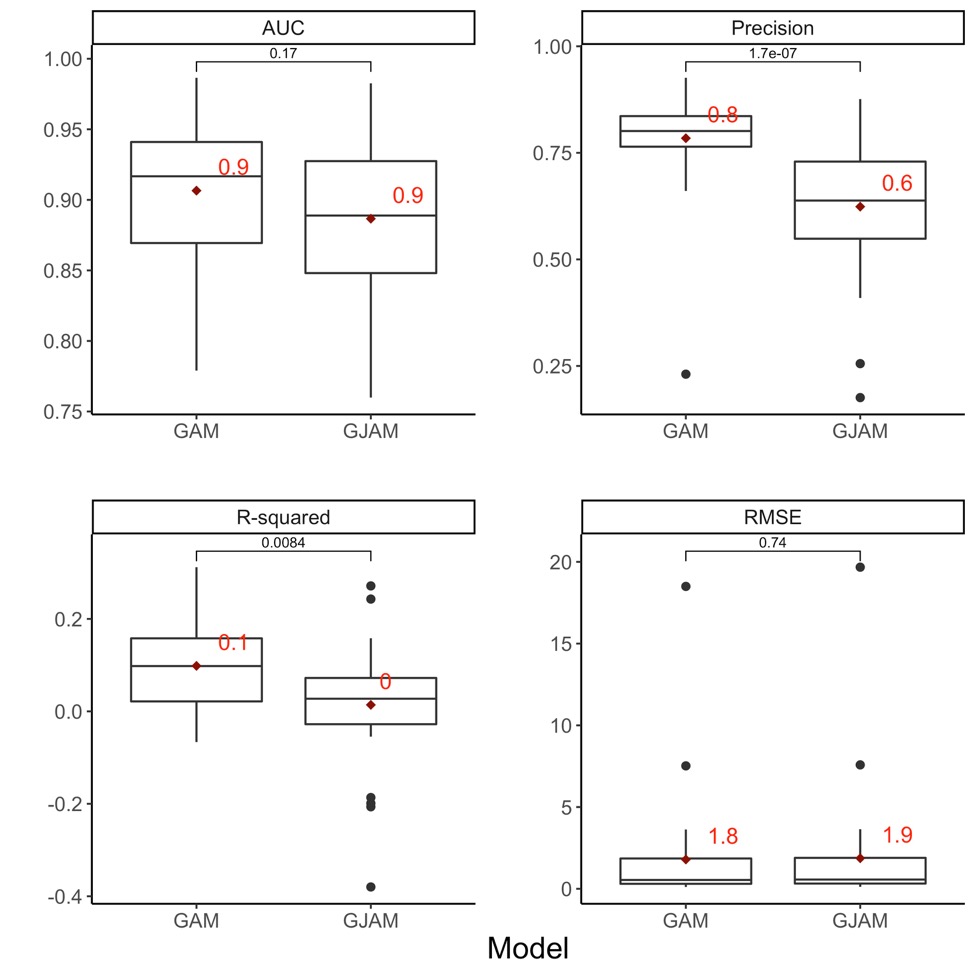


**Supplemental Figure S23. Model comparison between GJAM and GAMs in the fall.** AUC, Precision, R-squared, and RMSE comparison for out of sample prediction. Mean values are denoted in red. Brackets and numbers represent p-value. Whiskers represent 1.5* interquartile range. Box represents interquartile range as distance between first and third quartiles. Line represents median, red point represents mean, and black points represent outliers (outside of 1.5*IQR).


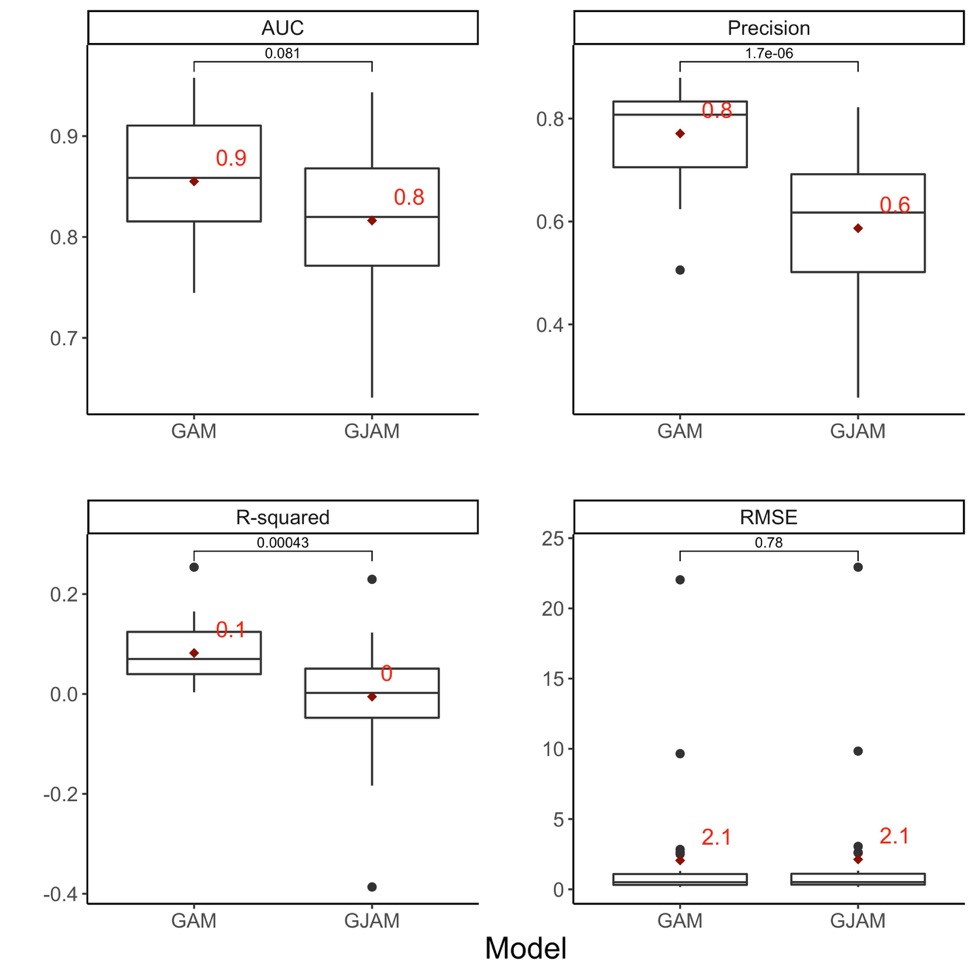


**Supplemental Figure S24. Model comparison between GJAM and GAMs in the spring.** AUC, Precision, R-squared, and RMSE comparison for out of sample prediction. Mean values are denoted in red. Brackets and numbers represent p-value. Whiskers represent 1.5* interquartile range. Box represents interquartile range as distance between first and third quartiles. Line represents median, red point represents mean, and black points represent outliers (outside of 1.5*IQR).


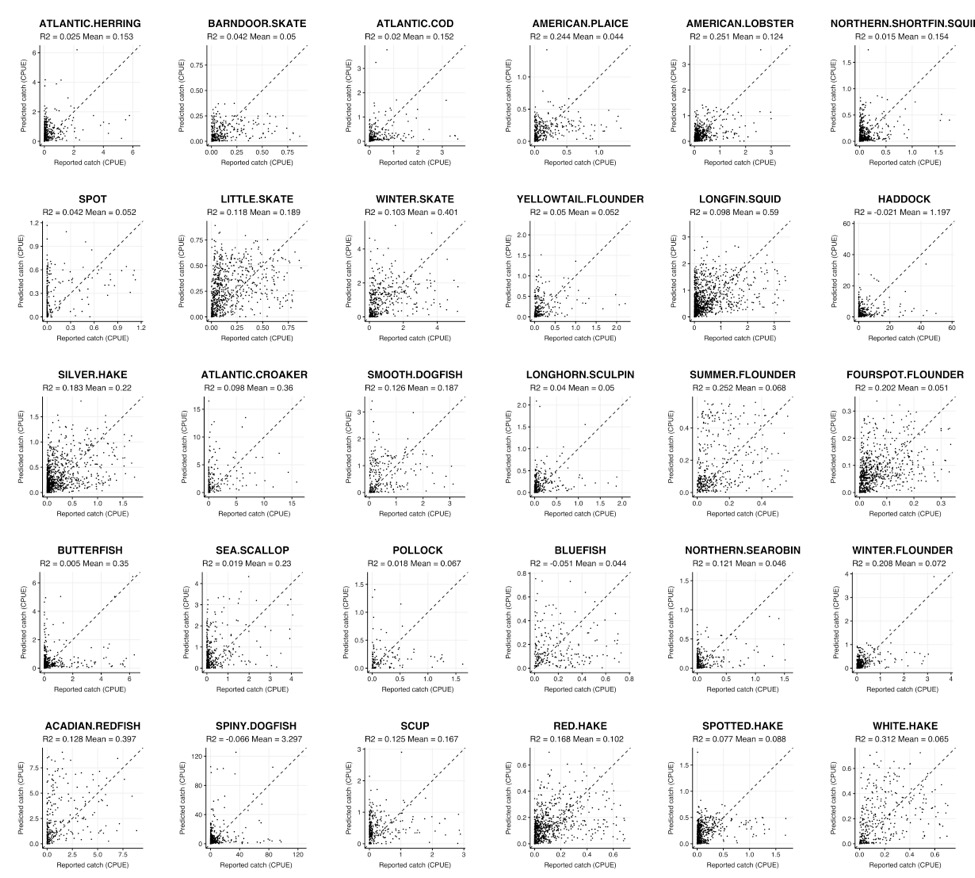


**Supplemental Figure S25. Out of sample prediction for fall species CPUE from GAM model.** RMSE and mean CPUE values noted in subtitle.


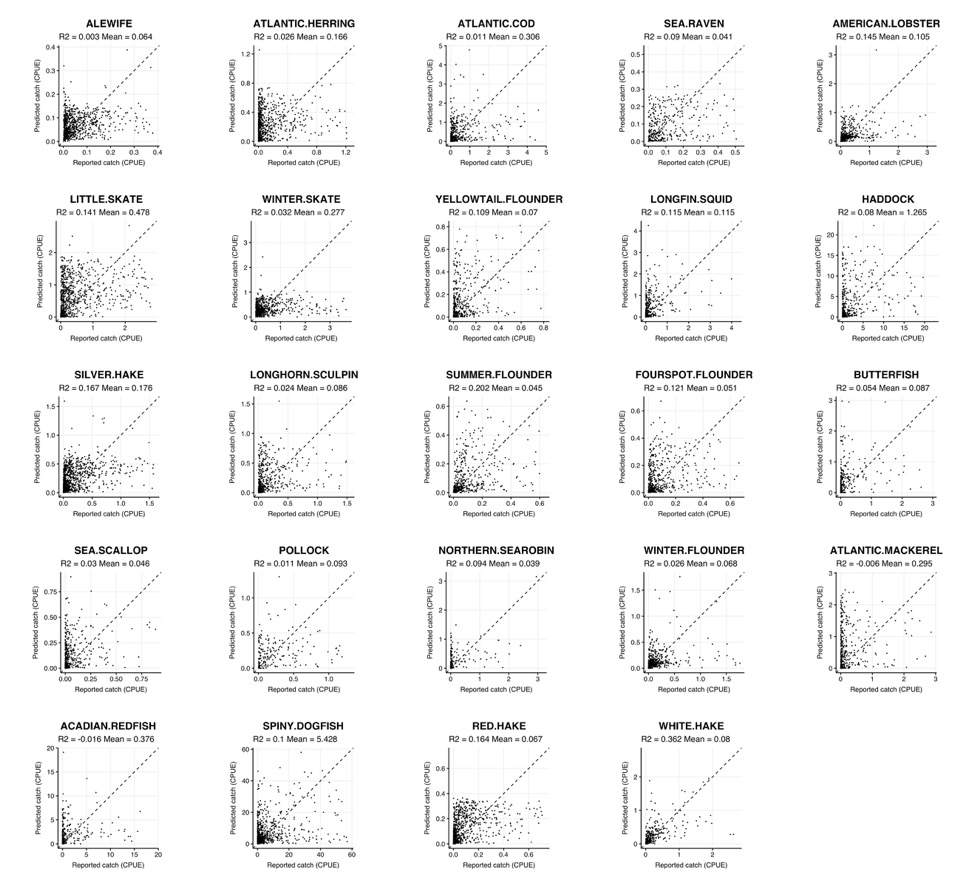


**Supplemental Figure S26. Out of sample prediction for spring species CPUE from GAM model.** RMSE and mean CPUE values noted in subtitle.

| names | GJAM RMSE | GJAM R^2^ | GJAM AUC | GJAM Precision | GAM RMSE | GAM R^2^ | GAM AUC | GAM Precision | Nonzero | mean cpue |
| --- | --- | --- | --- | --- | --- | --- | --- | --- | --- | --- |
| Atlantic herring | 1.84 | -0.19 | 0.91 | 0.63 | 1.67 | 0.02 | 0.95 | 0.85 | 1552 | 0.22 |
| barndoor skate | 0.33 | 0.01 | 0.76 | 0.26 | 0.33 | 0.04 | 0.85 | 0.69 | 981 | 0.04 |
| Atlantic cod | 2.36 | 0.01 | 0.90 | 0.41 | 2.35 | 0.02 | 0.92 | 0.78 | 784 | 0.13 |
| American plaice | 0.14 | 0.24 | 0.97 | 0.71 | 0.14 | 0.24 | 0.97 | 0.87 | 1220 | 0.05 |
| American lobster | 0.36 | 0.11 | 0.81 | 0.64 | 0.33 | 0.25 | 0.82 | 0.78 | 2133 | 0.13 |
| northern shortfin squid | 2.17 | 0.01 | 0.83 | 0.75 | 2.16 | 0.01 | 0.85 | 0.76 | 2566 | 0.09 |
| spot | 0.52 | 0.06 | 0.97 | 0.63 | 0.53 | 0.04 | 0.97 | 0.78 | 368 | 0.05 |
| little skate | 0.58 | 0.04 | 0.85 | 0.65 | 0.55 | 0.12 | 0.90 | 0.86 | 2490 | 0.17 |
| winter skate | 1.91 | 0.07 | 0.86 | 0.46 | 1.88 | 0.10 | 0.92 | 0.83 | 1391 | 0.37 |
| yellowtail flounder | 0.35 | -0.03 | 0.88 | 0.50 | 0.34 | 0.05 | 0.91 | 0.77 | 985 | 0.06 |
| longfin squid | 1.81 | 0.07 | 0.93 | 0.88 | 1.78 | 0.10 | 0.94 | 0.93 | 3651 | 0.54 |
| haddock | 7.58 | -0.04 | 0.84 | 0.53 | 7.52 | -0.02 | 0.87 | 0.75 | 1745 | 1.23 |
| silver hake | 0.55 | 0.11 | 0.85 | 0.83 | 0.53 | 0.18 | 0.87 | 0.87 | 3633 | 0.23 |
| Atlantic croaker | 3.20 | 0.05 | 0.98 | 0.74 | 3.13 | 0.10 | 0.98 | 0.83 | 497 | 0.34 |
| smooth dogfish | 1.03 | 0.02 | 0.91 | 0.46 | 0.97 | 0.13 | 0.93 | 0.74 | 854 | 0.19 |
| longhorn sculpin | 0.32 | -0.04 | 0.92 | 0.61 | 0.31 | 0.04 | 0.93 | 0.83 | 1130 | 0.05 |
| summer flounder | 0.22 | 0.16 | 0.93 | 0.67 | 0.21 | 0.25 | 0.96 | 0.86 | 1372 | 0.07 |
| fourspot flounder | 0.12 | 0.07 | 0.84 | 0.72 | 0.11 | 0.20 | 0.86 | 0.80 | 2523 | 0.05 |
| butterfish | 3.65 | -0.01 | 0.76 | 0.71 | 3.63 | 0.00 | 0.78 | 0.81 | 3182 | 0.28 |
| sea scallop | 1.67 | 0.02 | 0.85 | 0.64 | 1.67 | 0.02 | 0.87 | 0.79 | 1908 | 0.23 |
| pollock | 0.74 | -0.02 | 0.88 | 0.18 | 0.73 | 0.02 | 0.89 | 0.23 | 533 | 0.08 |
| bluefish | 0.24 | -0.38 | 0.92 | 0.52 | 0.21 | -0.05 | 0.92 | 0.66 | 768 | 0.05 |
| northern searobin | 0.32 | 0.03 | 0.92 | 0.59 | 0.30 | 0.12 | 0.92 | 0.76 | 1241 | 0.04 |
| winter flounder | 0.32 | 0.03 | 0.87 | 0.63 | 0.29 | 0.21 | 0.89 | 0.72 | 1276 | 0.09 |
| Acadian redfish | 2.29 | -0.20 | 0.98 | 0.78 | 1.96 | 0.13 | 0.99 | 0.88 | 1147 | 0.51 |
| spiny dogfish | 19.68 | -0.21 | 0.82 | 0.64 | 18.50 | -0.07 | 0.84 | 0.79 | 2364 | 3.77 |
| scup | 0.84 | 0.08 | 0.96 | 0.73 | 0.81 | 0.13 | 0.96 | 0.83 | 1060 | 0.15 |
| red hake | 0.28 | 0.12 | 0.86 | 0.76 | 0.27 | 0.17 | 0.87 | 0.83 | 2751 | 0.10 |
| spotted hake | 0.47 | -0.05 | 0.91 | 0.78 | 0.44 | 0.08 | 0.92 | 0.84 | 2132 | 0.09 |
| white hake | 0.19 | 0.27 | 0.94 | 0.70 | 0.18 | 0.31 | 0.94 | 0.80 | 1434 | 0.07 |

**Supplemental Table 1. Fall Model results.** RMSE, R^2^, AUC, precision for GJAM and GAM models as well as total non-zero rows and mean CPUE for the entire fall dataset.

| names | GJAM RMSE | GJAM R^2^ | GJAM AUC | GJAM Precision | GAM RMSE | GAM R^2^ | GAM AUC | GAM Precision | Nonzero | mean cpue |
| --- | --- | --- | --- | --- | --- | --- | --- | --- | --- | --- |
| alewife | 0.37 | -0.04 | 0.75 | 0.61 | 0.36 | 0.00 | 0.78 | 0.82 | 2778 | 0.06 |
| Atlantic herring | 0.85 | -0.05 | 0.68 | 0.69 | 0.82 | 0.03 | 0.74 | 0.74 | 3407 | 0.17 |
| Atlantic cod | 2.62 | 0.03 | 0.84 | 0.52 | 2.64 | 0.01 | 0.86 | 0.71 | 1563 | 0.25 |
| sea raven | 0.18 | 0.03 | 0.77 | 0.37 | 0.17 | 0.09 | 0.82 | 0.51 | 1376 | 0.05 |
| American lobster | 0.33 | 0.00 | 0.79 | 0.60 | 0.31 | 0.15 | 0.81 | 0.75 | 1986 | 0.12 |
| little skate | 1.33 | 0.10 | 0.83 | 0.70 | 1.30 | 0.14 | 0.85 | 0.81 | 3182 | 0.47 |
| winter skate | 0.95 | -0.04 | 0.72 | 0.46 | 0.91 | 0.03 | 0.77 | 0.69 | 2369 | 0.27 |
| yellowtail flounder | 0.43 | 0.03 | 0.81 | 0.57 | 0.41 | 0.11 | 0.87 | 0.75 | 1624 | 0.07 |
| longfin squid | 0.78 | 0.07 | 0.90 | 0.76 | 0.76 | 0.11 | 0.92 | 0.87 | 2215 | 0.13 |
| haddock | 9.84 | 0.01 | 0.85 | 0.57 | 9.51 | 0.08 | 0.91 | 0.82 | 1815 | 1.21 |
| silver hake | 0.48 | 0.11 | 0.79 | 0.82 | 0.46 | 0.17 | 0.82 | 0.85 | 4324 | 0.16 |
| longhorn sculpin | 0.55 | 0.06 | 0.88 | 0.72 | 0.56 | 0.02 | 0.94 | 0.88 | 2000 | 0.09 |
| summer flounder | 0.17 | 0.12 | 0.91 | 0.62 | 0.16 | 0.20 | 0.92 | 0.83 | 1749 | 0.05 |
| fourspot flounder | 0.21 | -0.06 | 0.78 | 0.65 | 0.19 | 0.12 | 0.88 | 0.86 | 2084 | 0.05 |
| butterfish | 0.73 | -0.18 | 0.91 | 0.62 | 0.65 | 0.05 | 0.92 | 0.82 | 1488 | 0.1 |
| sea scallop | 0.36 | -0.06 | 0.77 | 0.43 | 0.34 | 0.03 | 0.83 | 0.69 | 1782 | 0.06 |
| pollock | 1.03 | -0.02 | 0.85 | 0.28 | 1.02 | 0.01 | 0.85 | 0.62 | 701 | 0.09 |
| northern searobin | 0.33 | -0.39 | 0.84 | 0.43 | 0.27 | 0.09 | 0.87 | 0.68 | 1273 | 0.08 |
| winter flounder | 0.33 | 0.00 | 0.86 | 0.65 | 0.32 | 0.03 | 0.89 | 0.79 | 1706 | 0.07 |
| Atlantic mackerel | 2.60 | -0.06 | 0.64 | 0.26 | 2.53 | -0.01 | 0.74 | 0.69 | 1784 | 0.36 |
| Acadian redfish | 3.06 | -0.09 | 0.94 | 0.65 | 2.95 | -0.02 | 0.96 | 0.80 | 1179 | 0.4 |
| spiny dogfish | 22.93 | -0.01 | 0.77 | 0.67 | 21.67 | 0.10 | 0.82 | 0.85 | 3527 | 5.9 |
| red hake | 0.22 | 0.05 | 0.78 | 0.75 | 0.21 | 0.16 | 0.79 | 0.84 | 3146 | 0.07 |
| white hake | 0.40 | 0.23 | 0.94 | 0.71 | 0.36 | 0.36 | 0.95 | 0.82 | 1370 | 0.06 |

**Supplemental Table 2. Spring model results.** RMSE, R^2^, AUC, precision for GJAM and GAM models as well as total non-zero rows and mean CPUE for the entire spring dataset.
