# Supplementary material for: Jointly modeling marine species to inform the effects of environmental change on an ecological community in the Northwest Atlantic
Source: Sci Rep. 2022 Jan 7;12:132. doi: 10.1038/s41598-021-04110-0 (PMC8742080; doi:10.1038/s41598-021-04110-0)

**Supplemental Figure S27-57. Spatial and temporal autocorrelation in the fall.** Spatial pattern of residuals in 2015 (left), semi-variogram for each direction in 2015 (90 = east, 135 = south east, 0 = north, 45 = north-east), and partial autocorrelation function plot for 1000 days (starting in 1998).
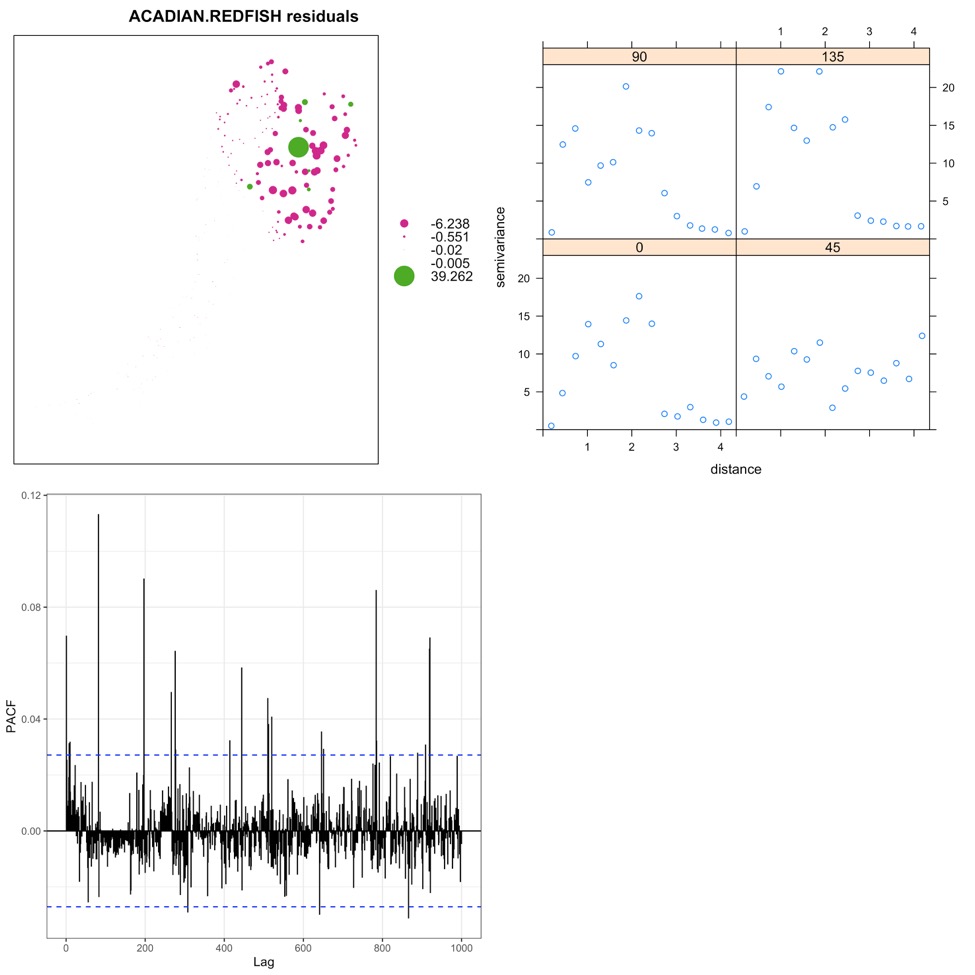

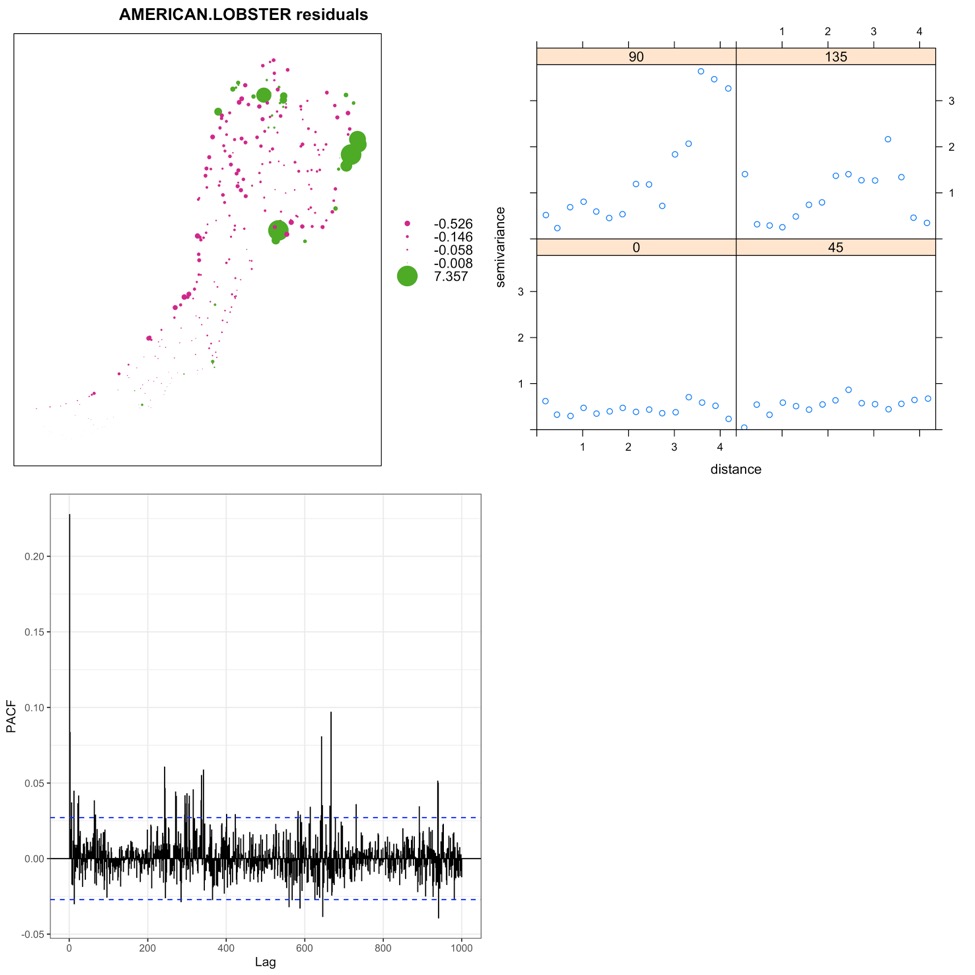

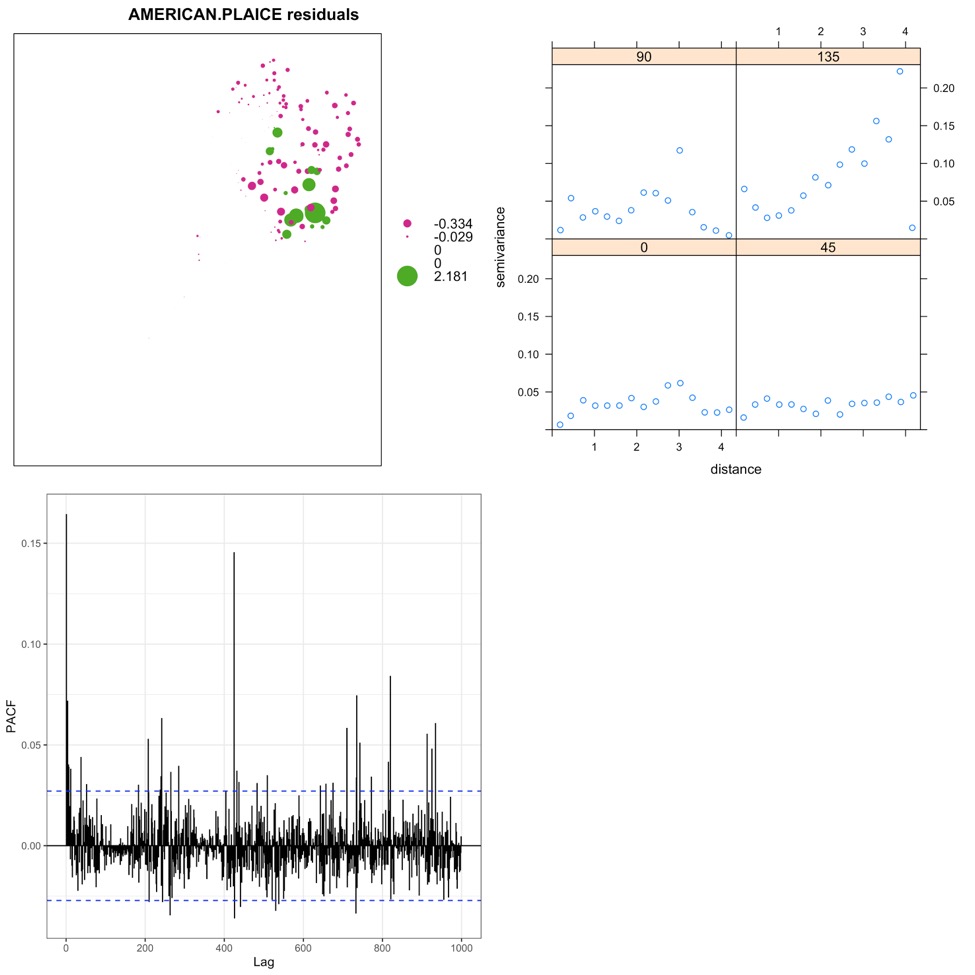

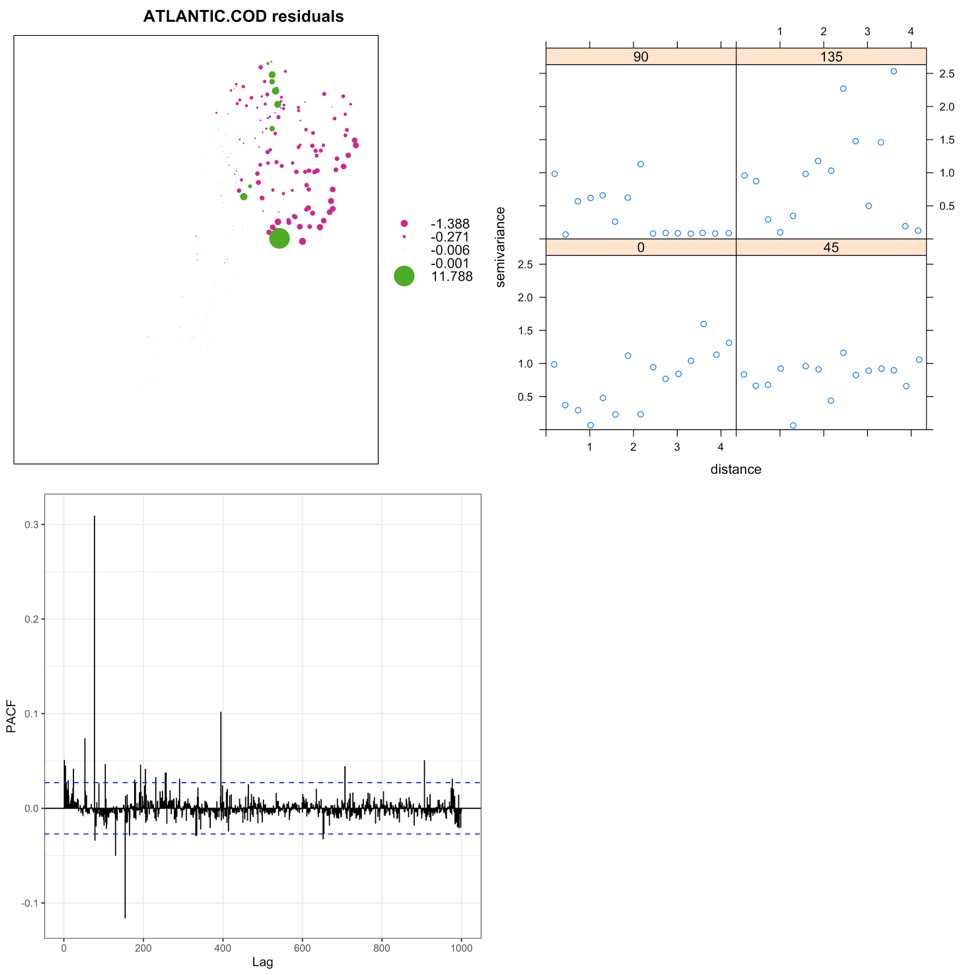

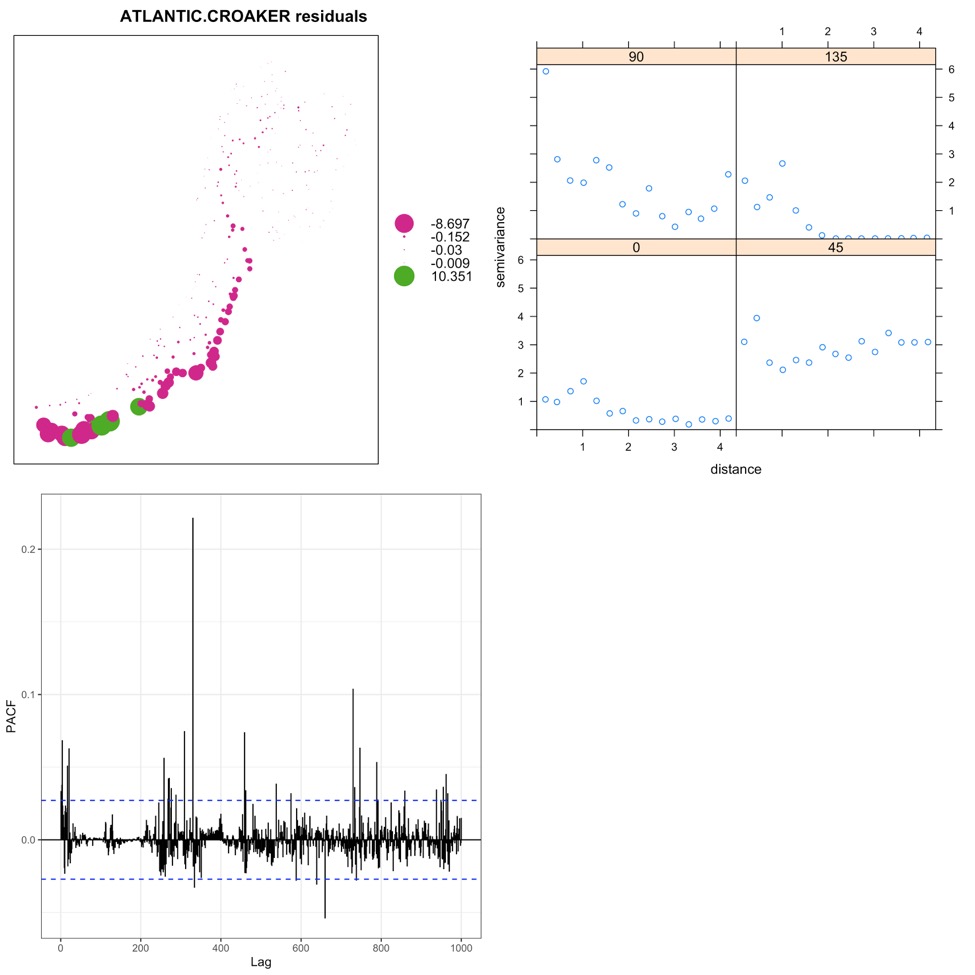

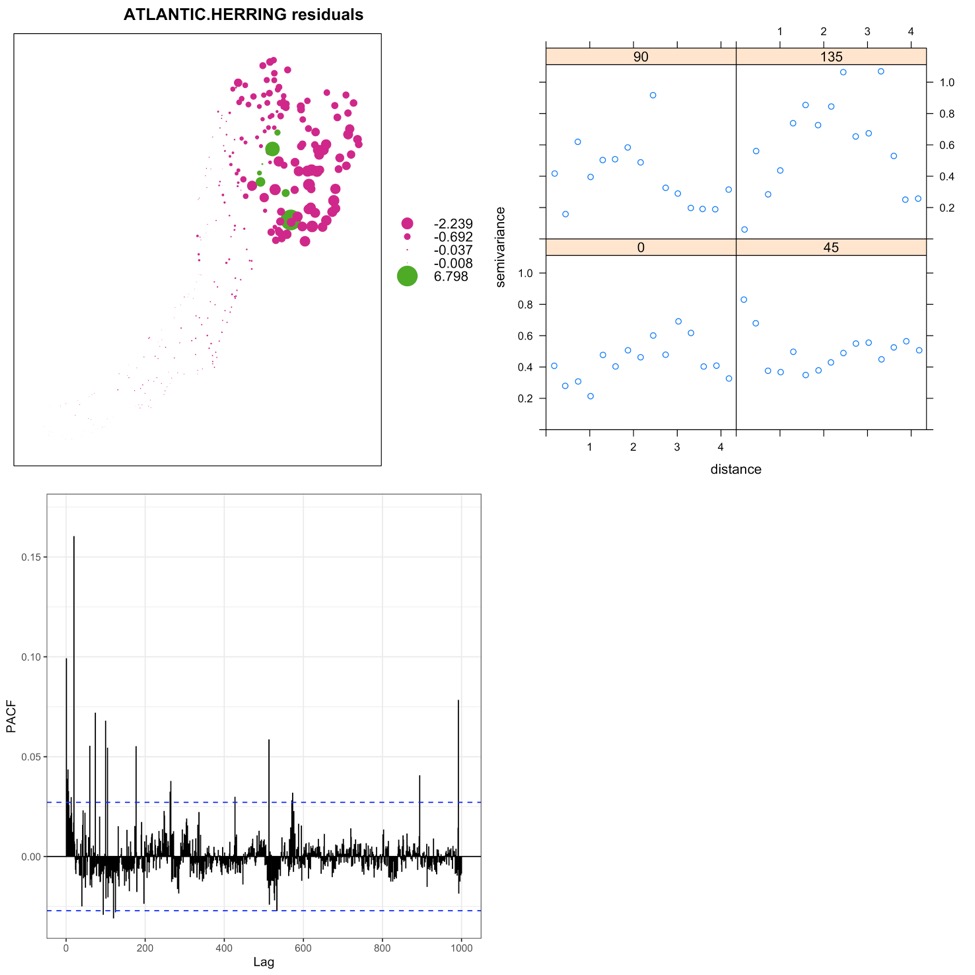

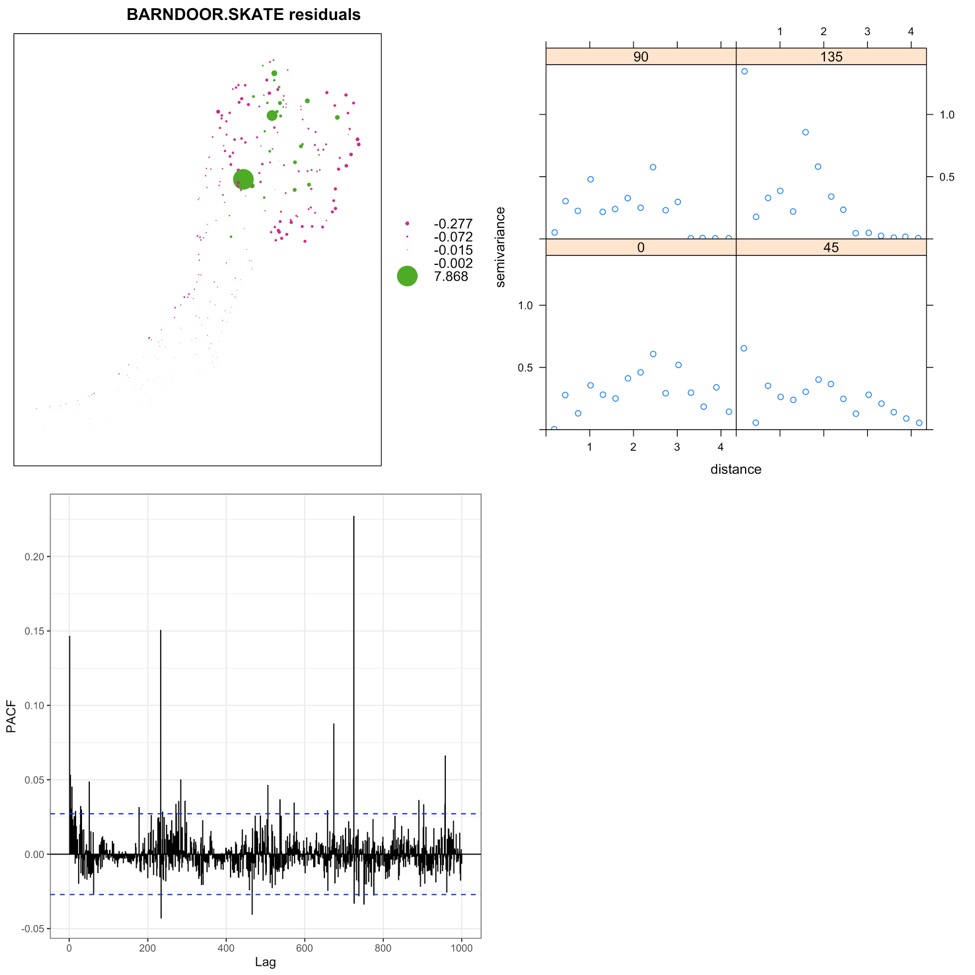

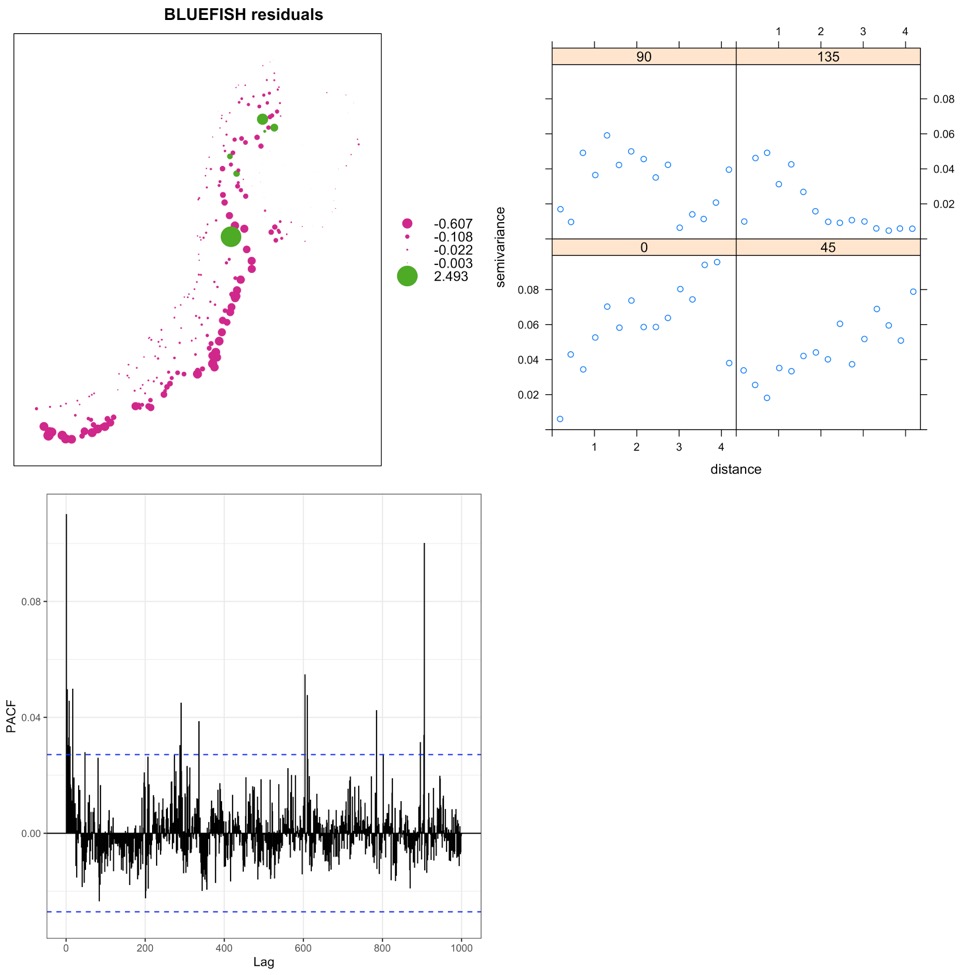

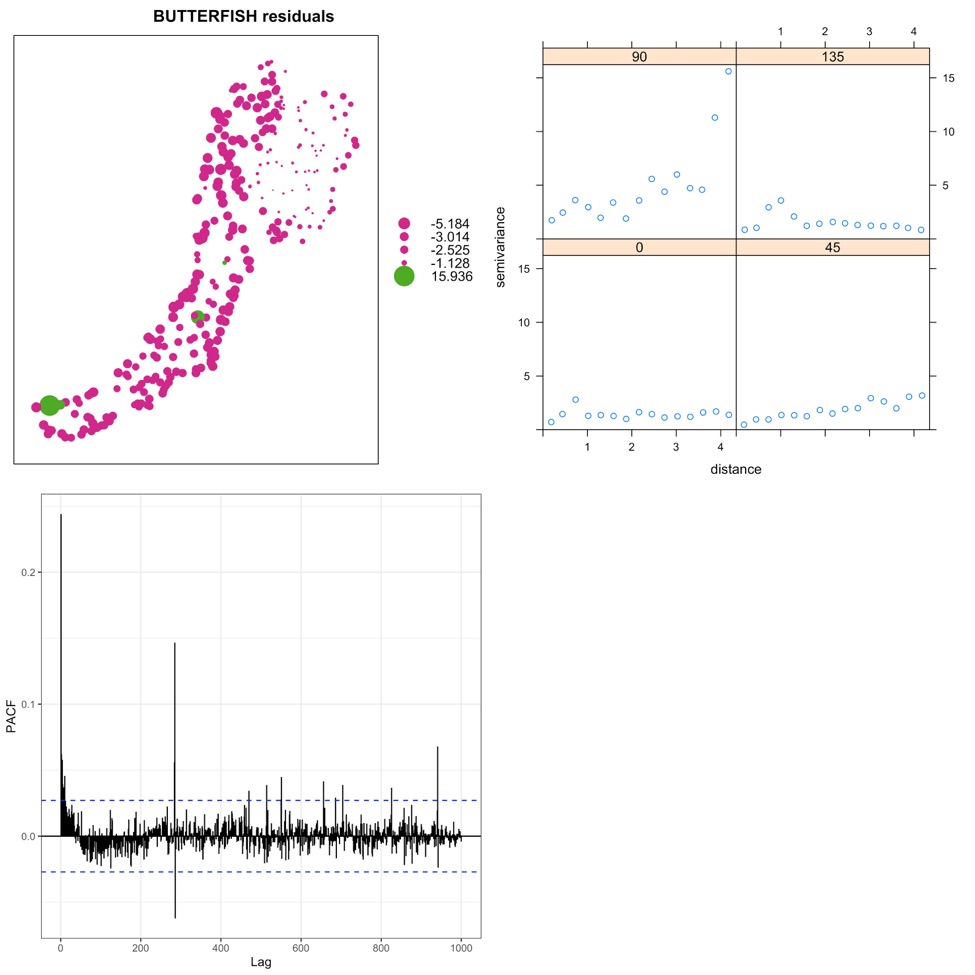

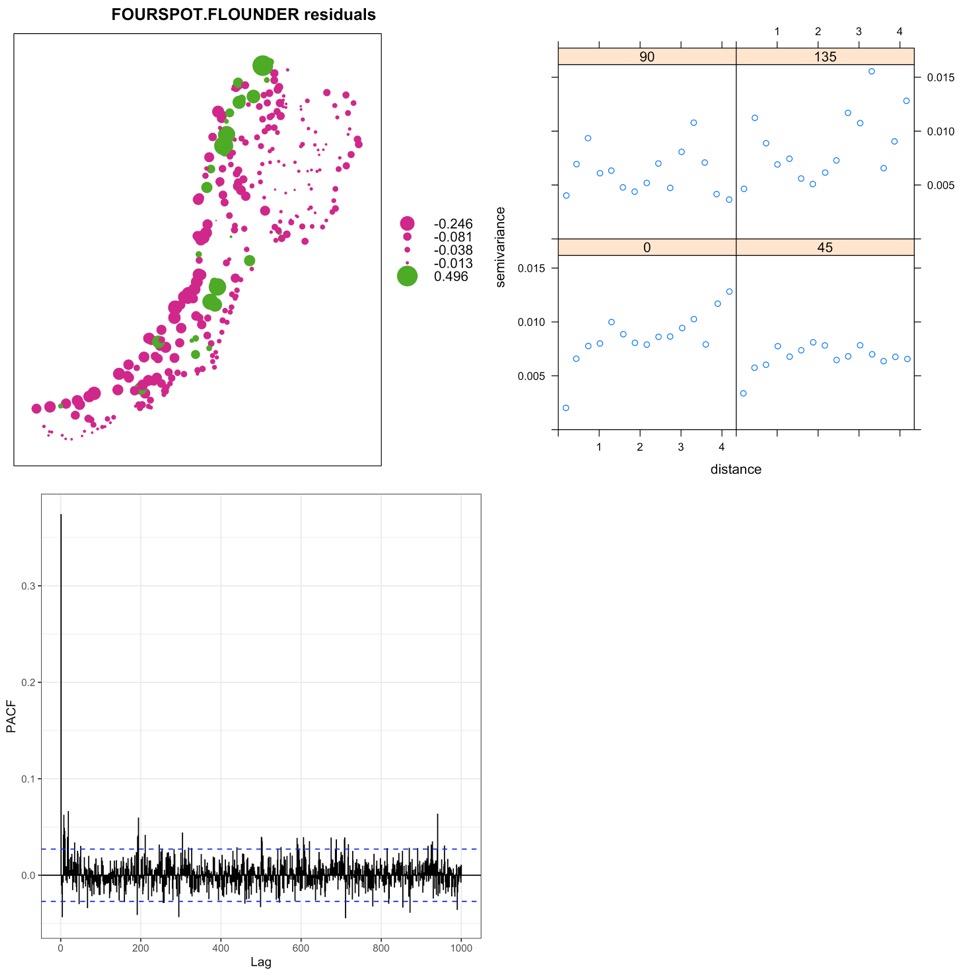

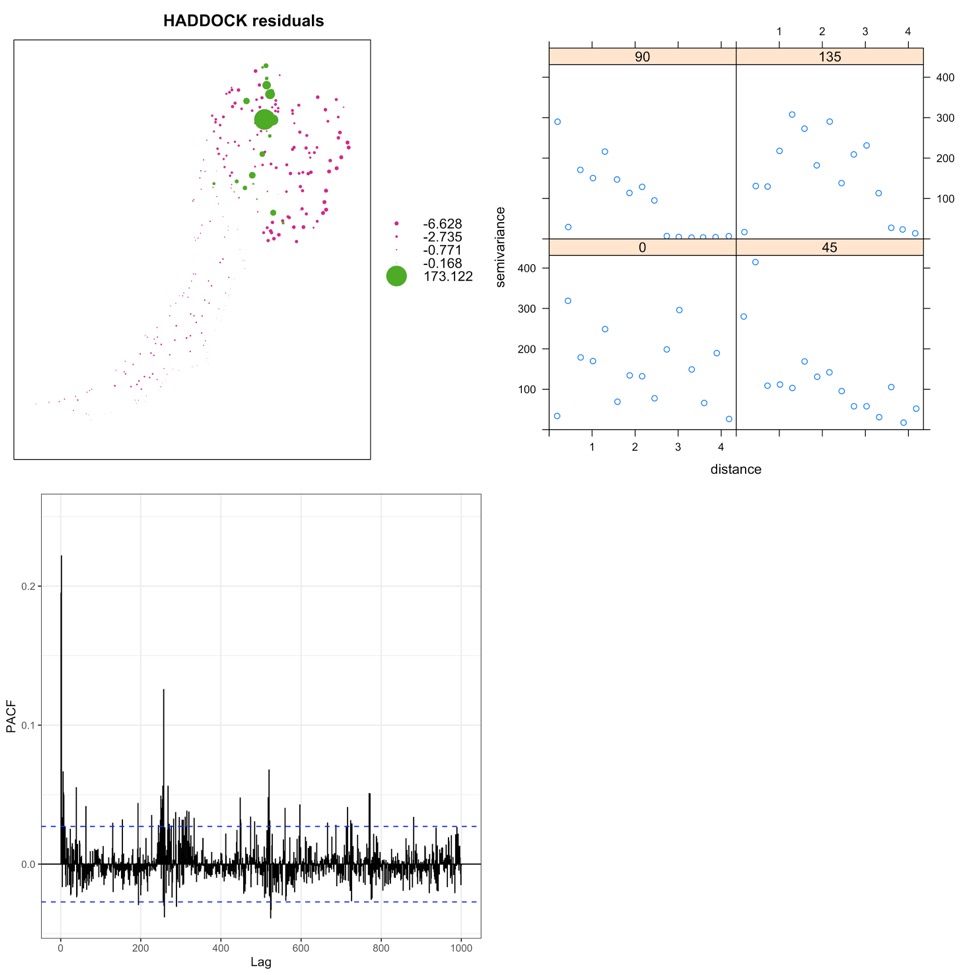

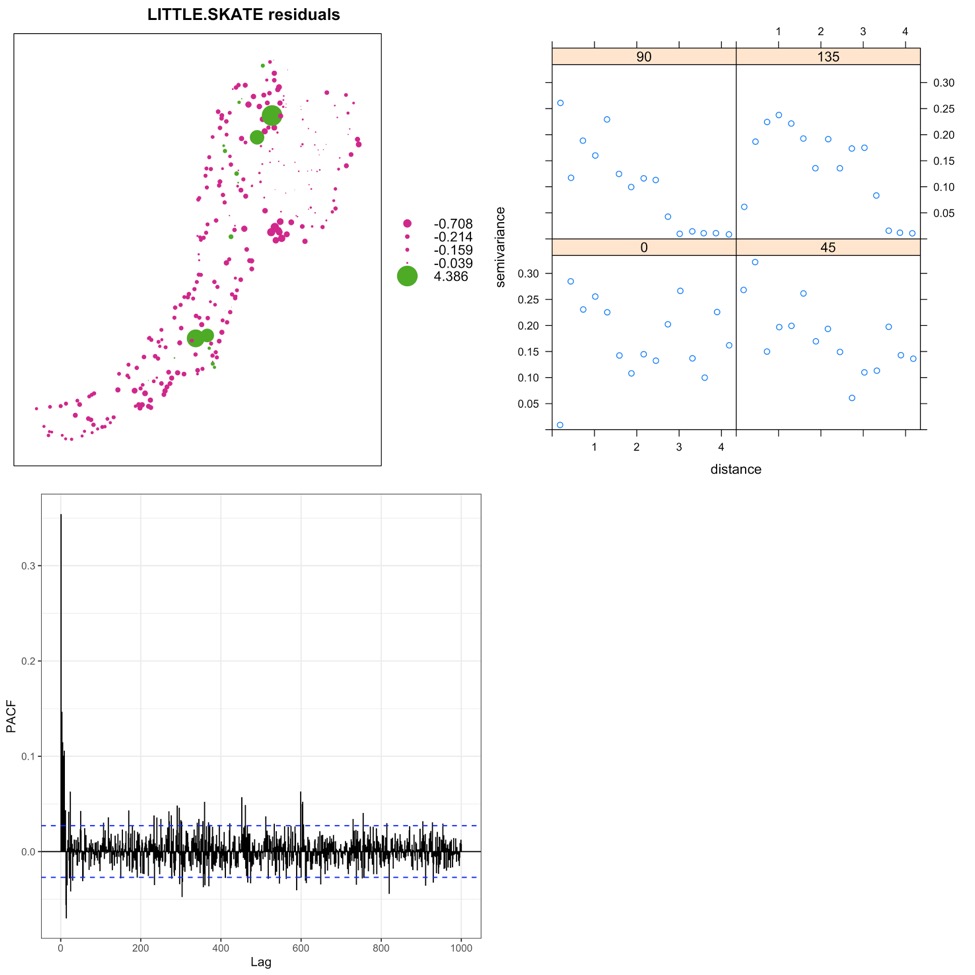

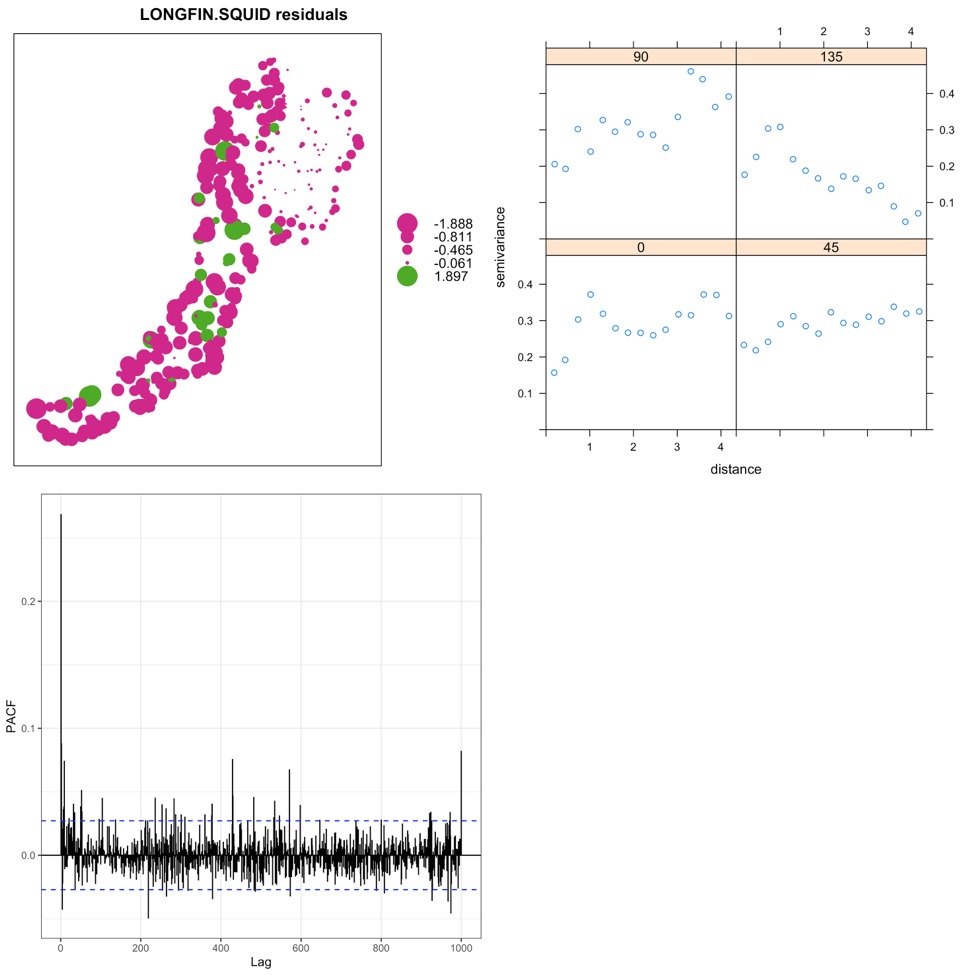

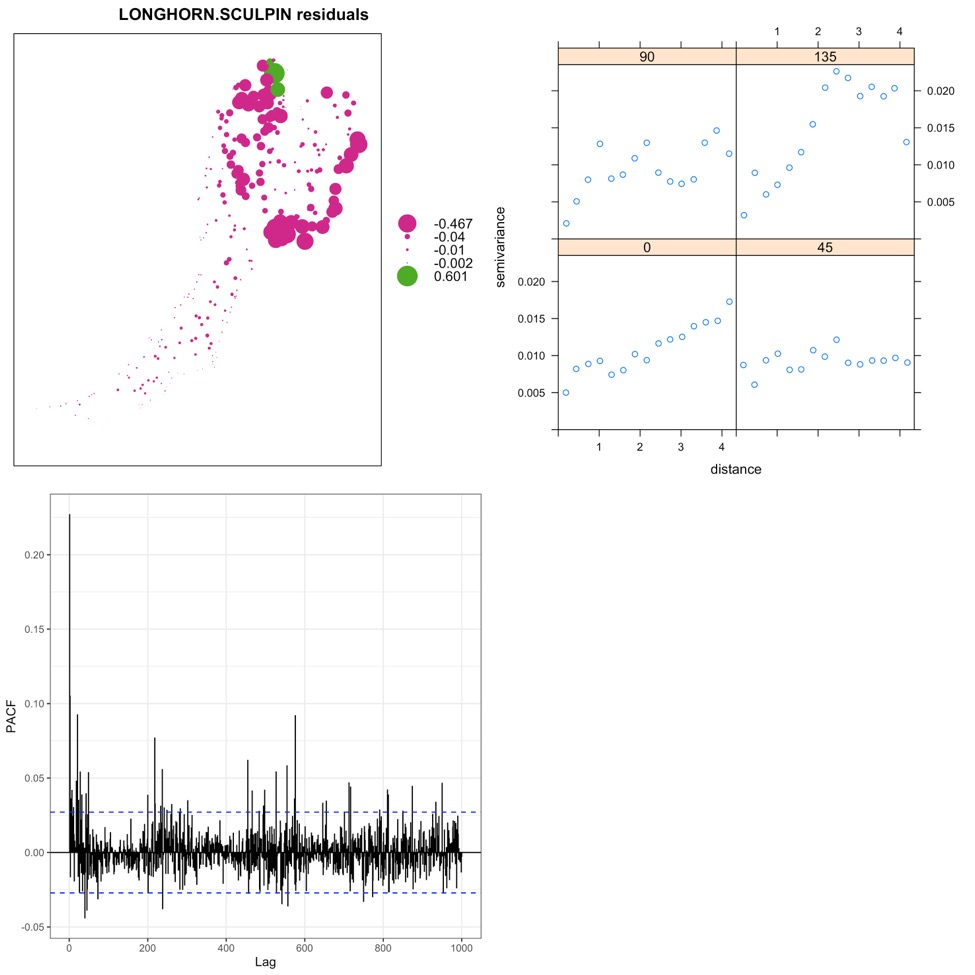

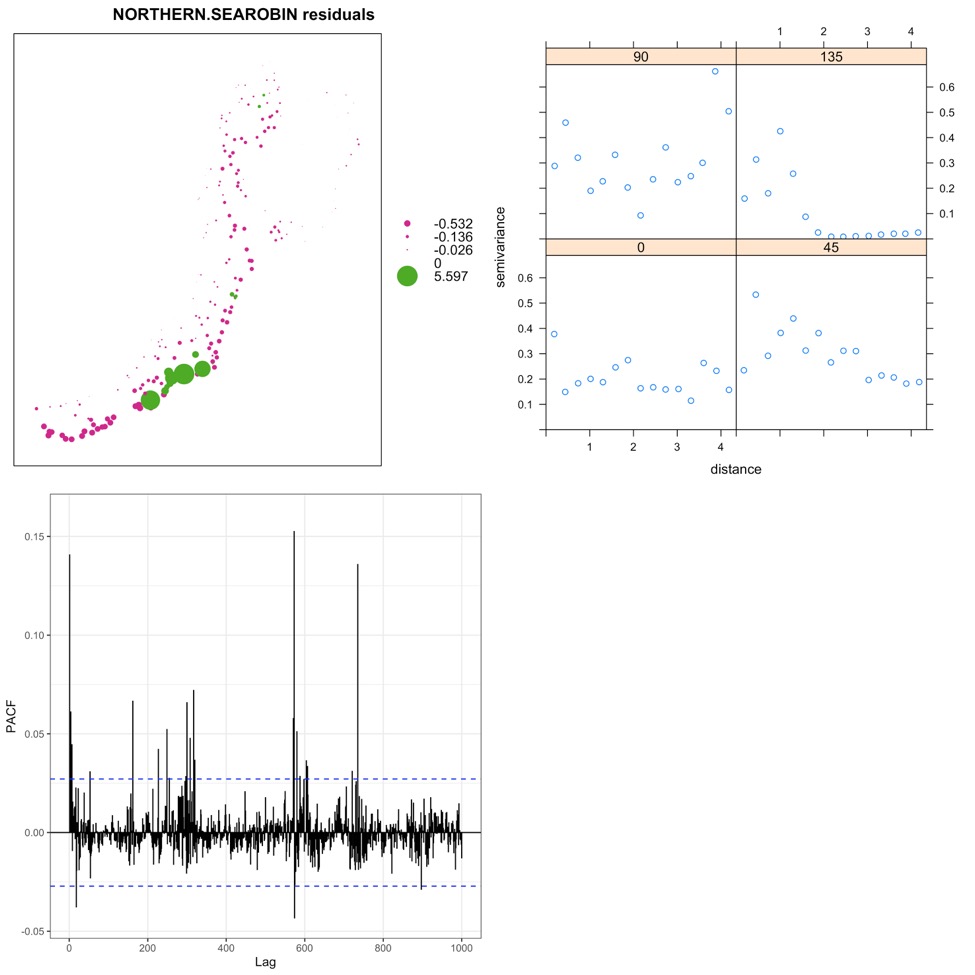

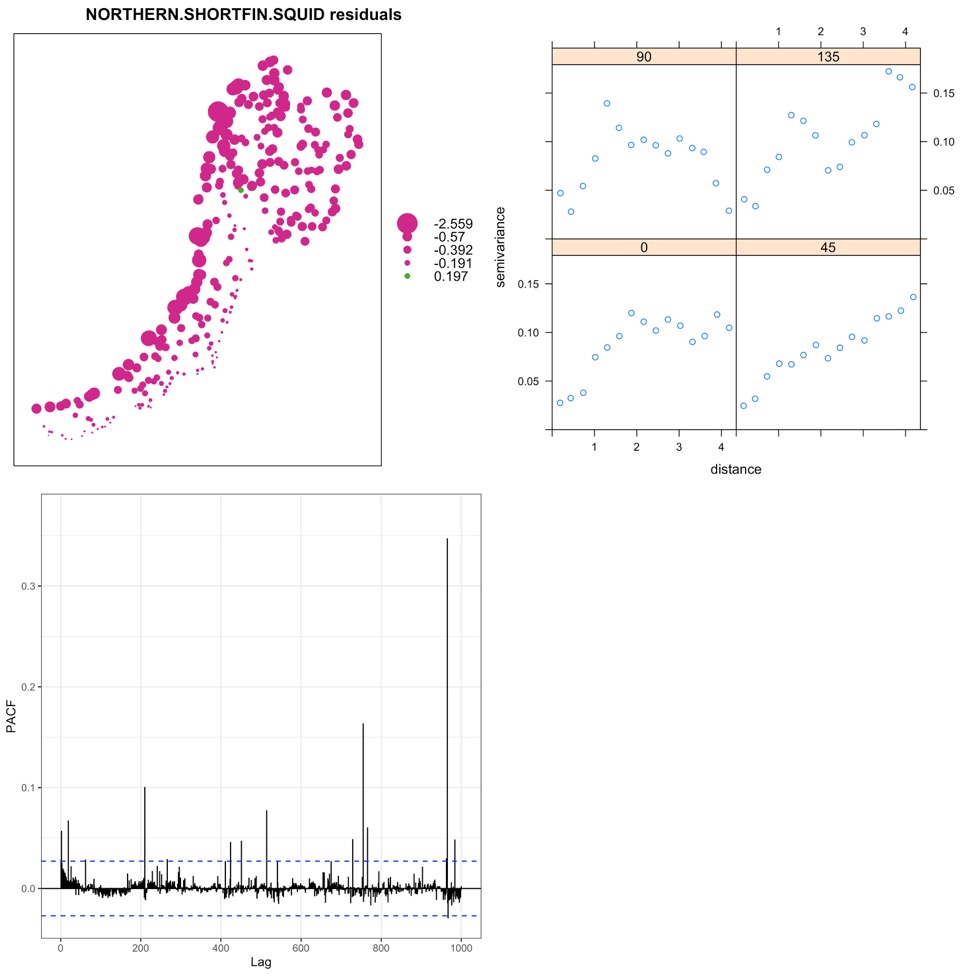

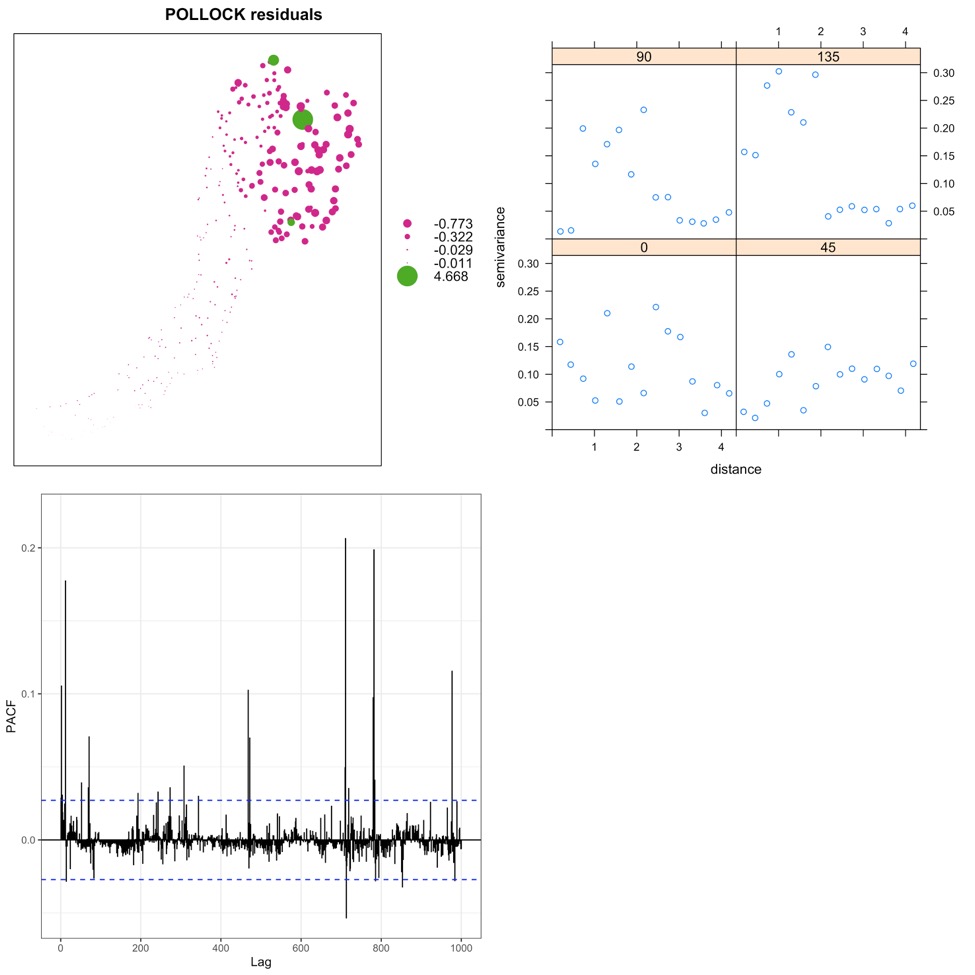

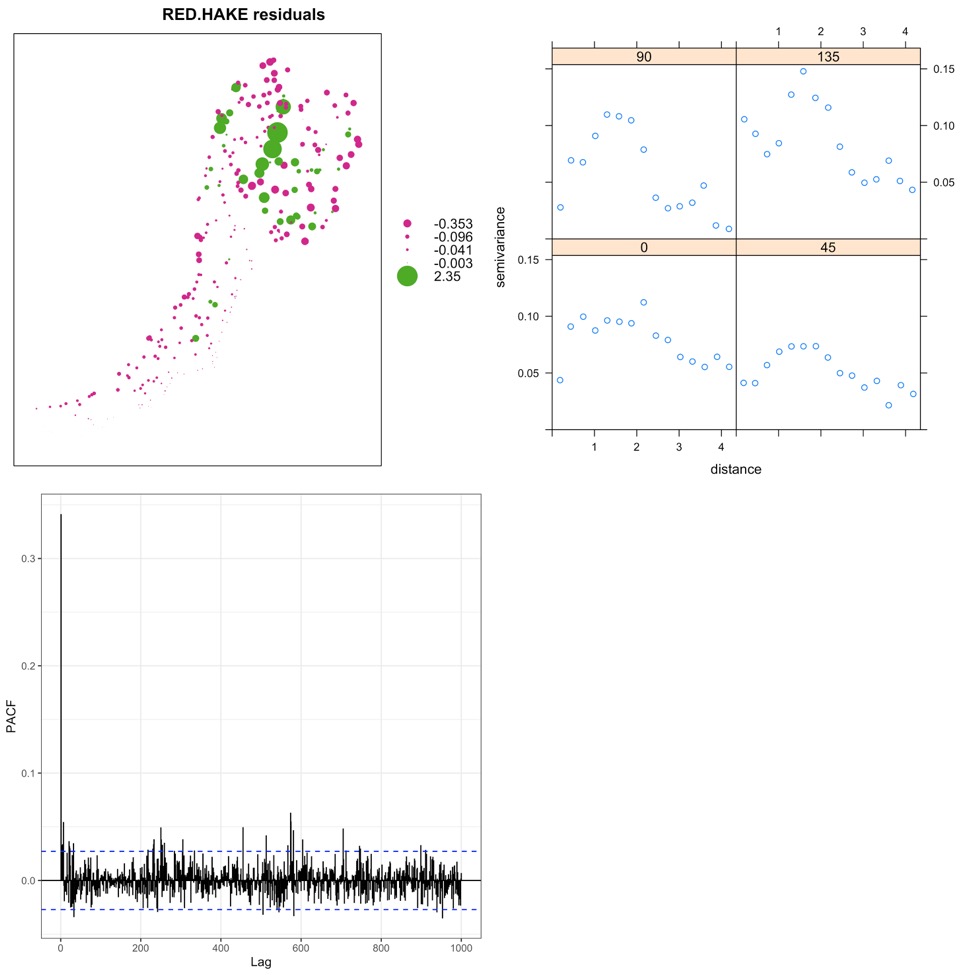

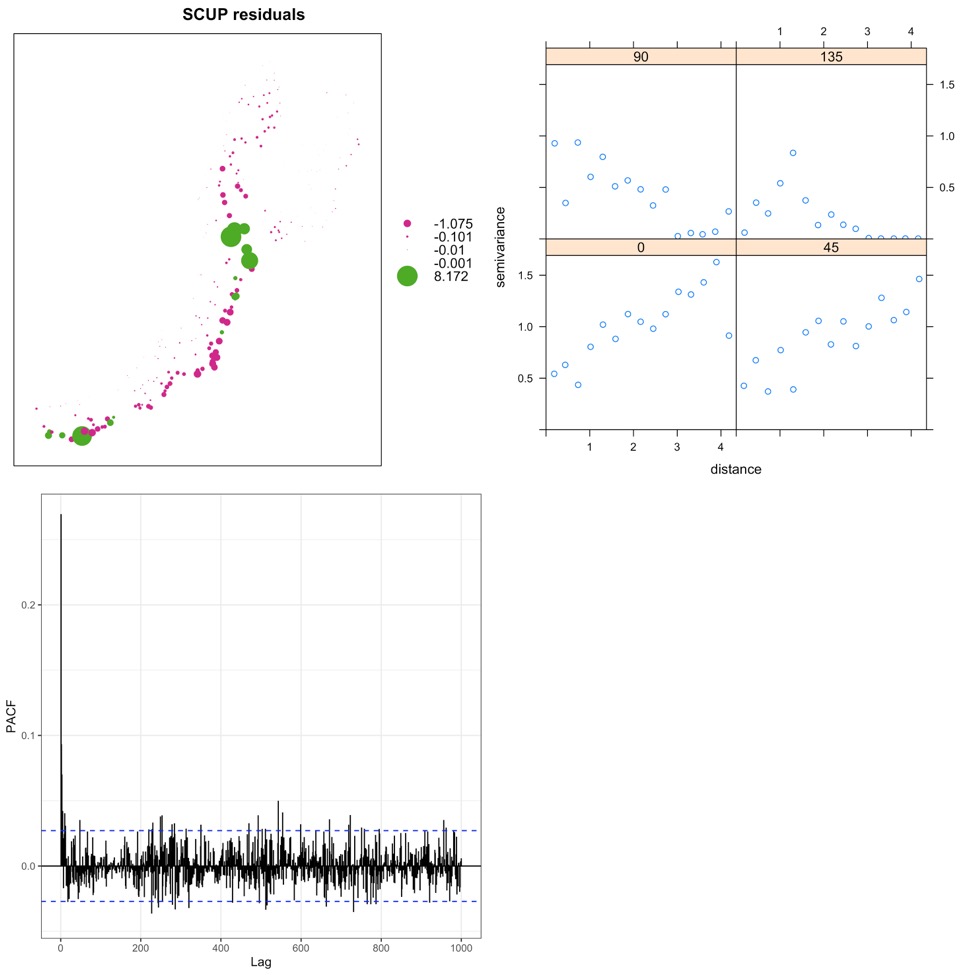

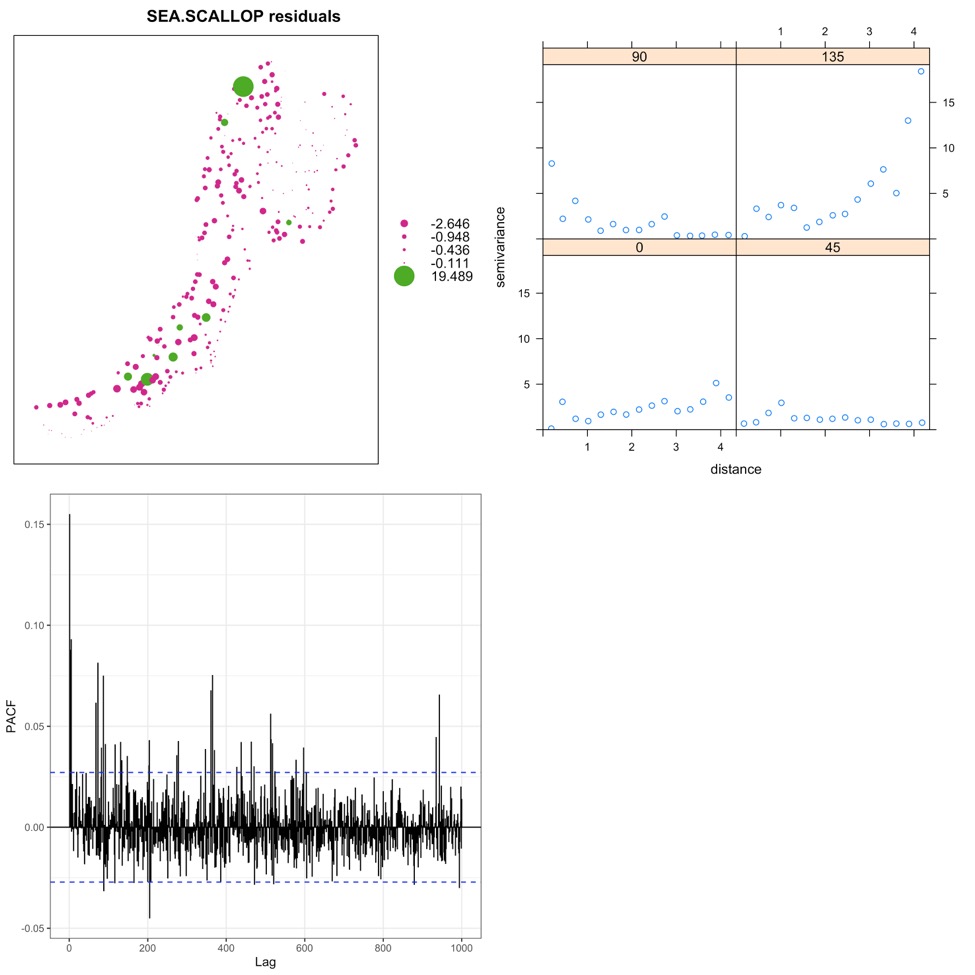

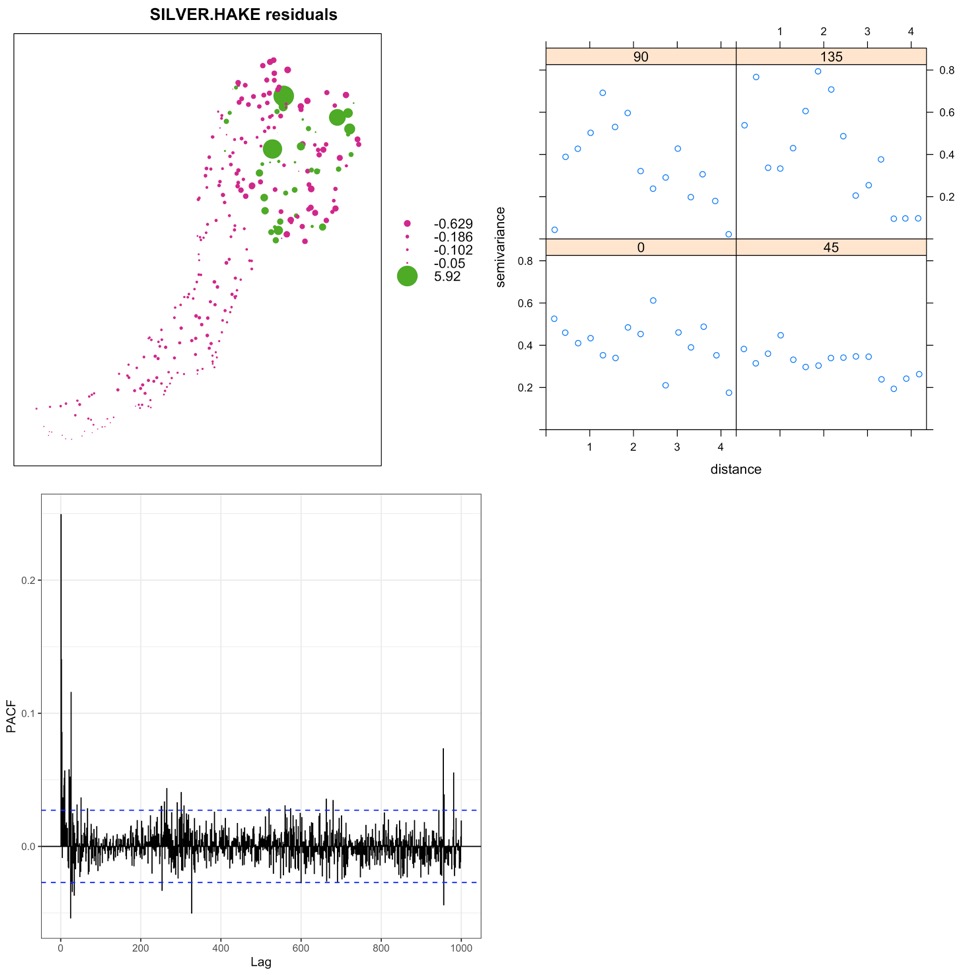

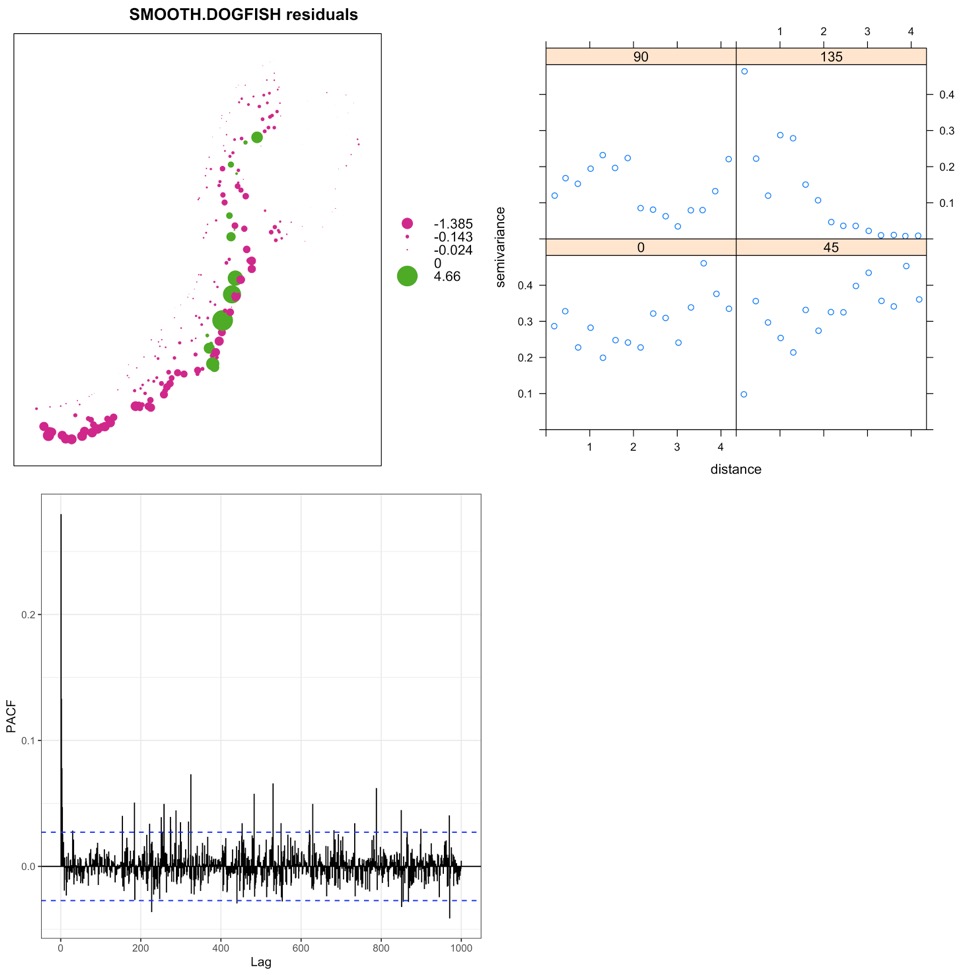

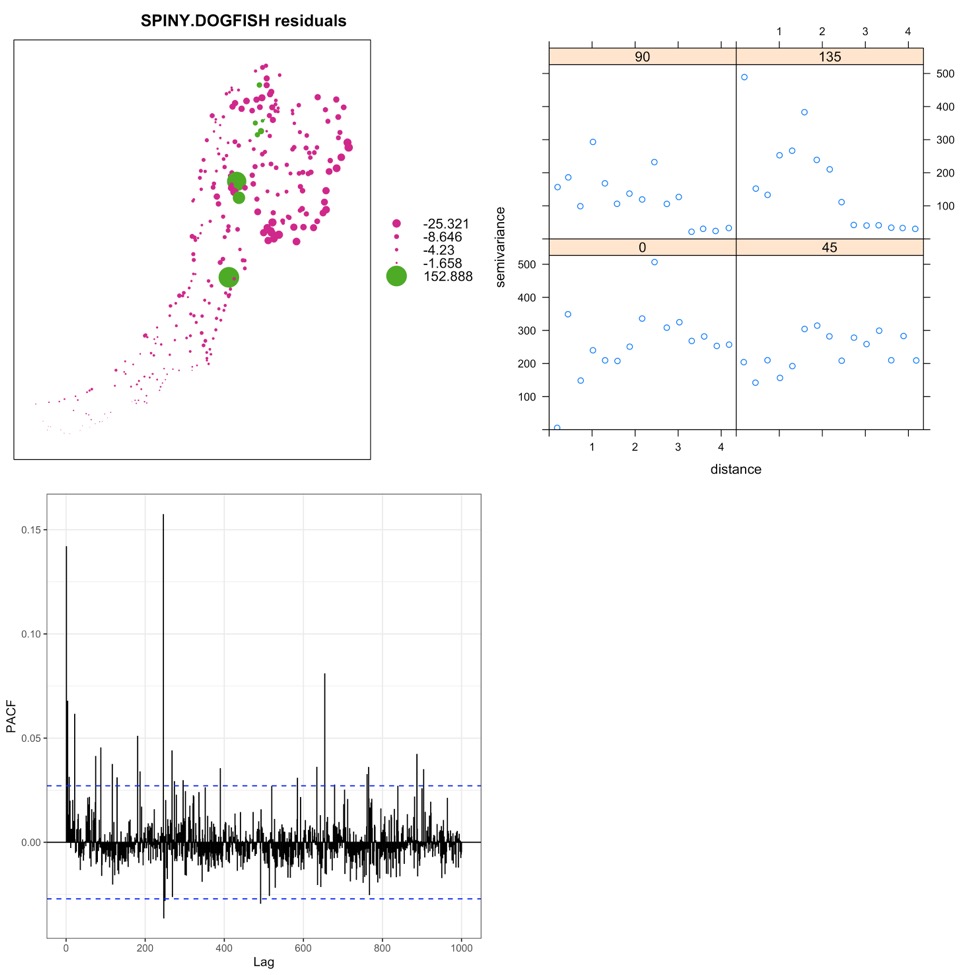

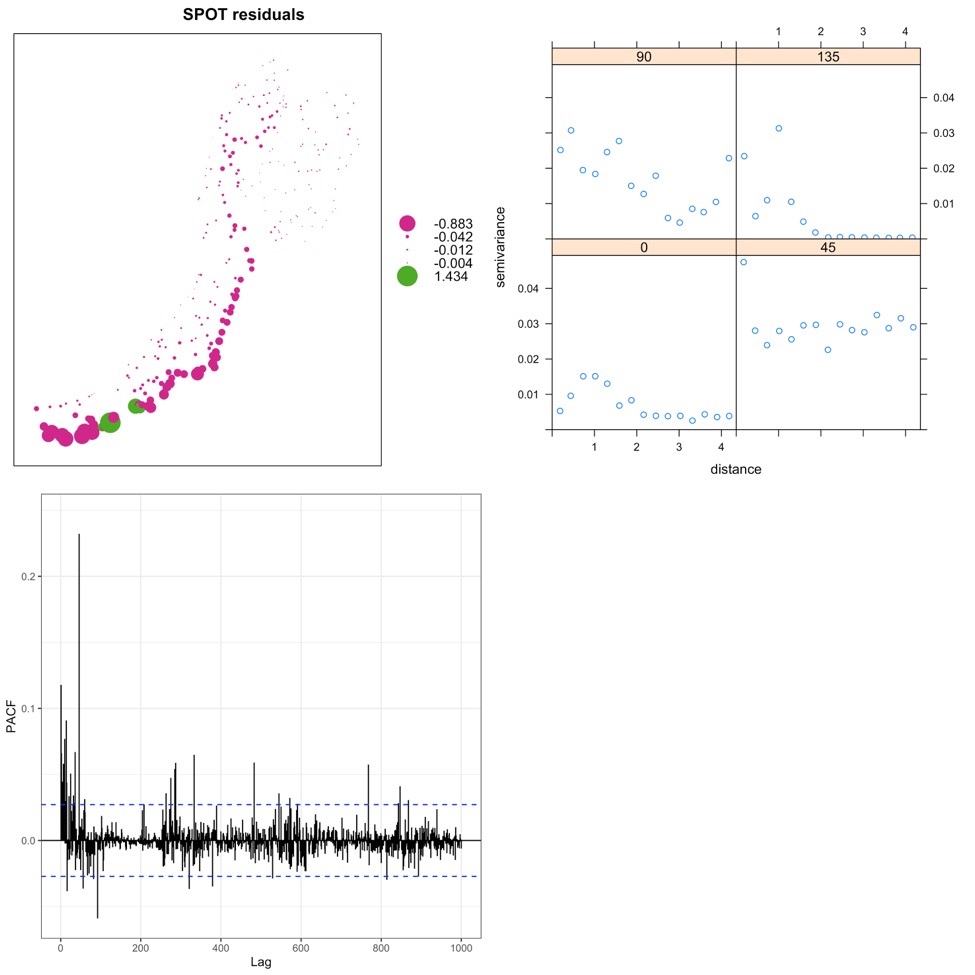

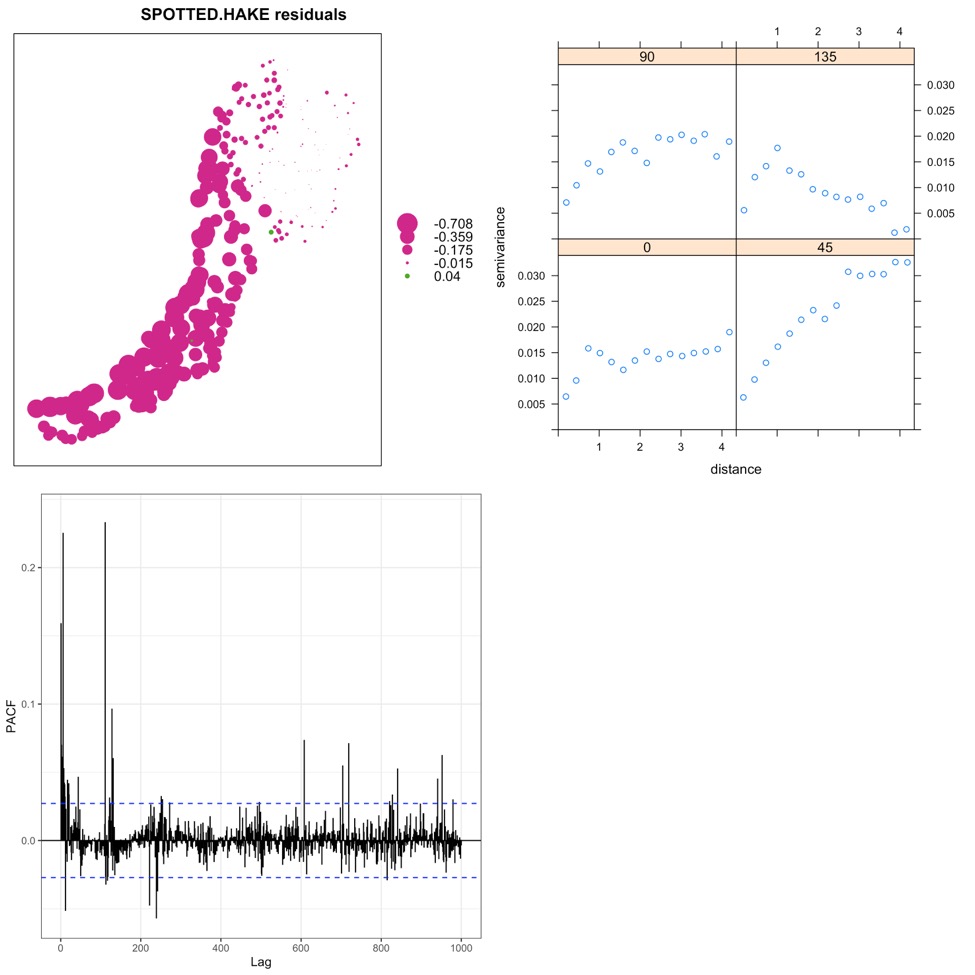

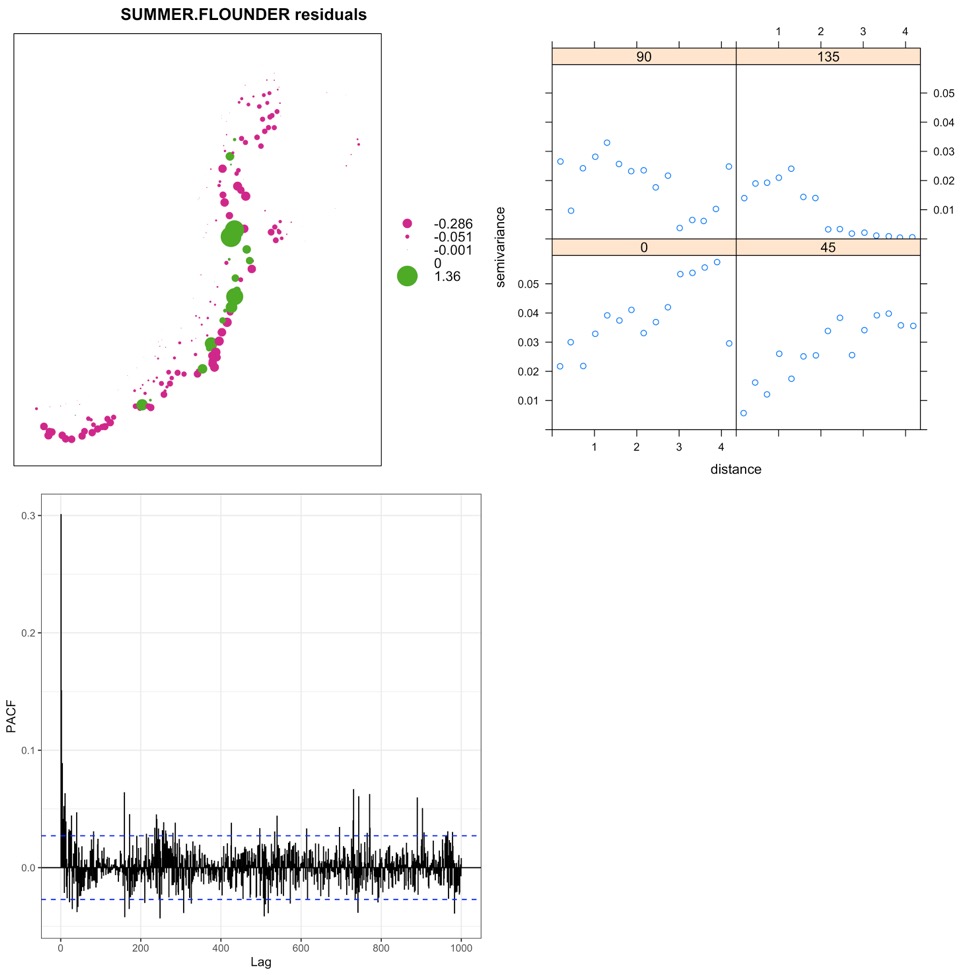

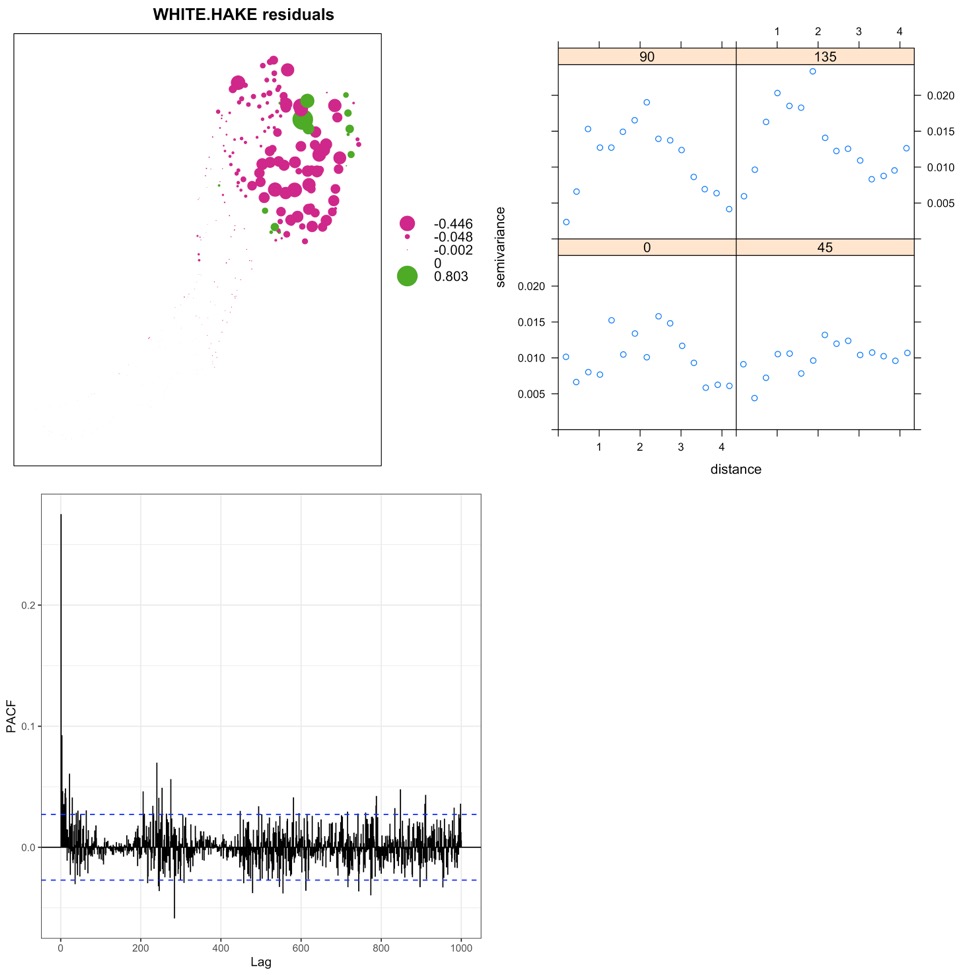

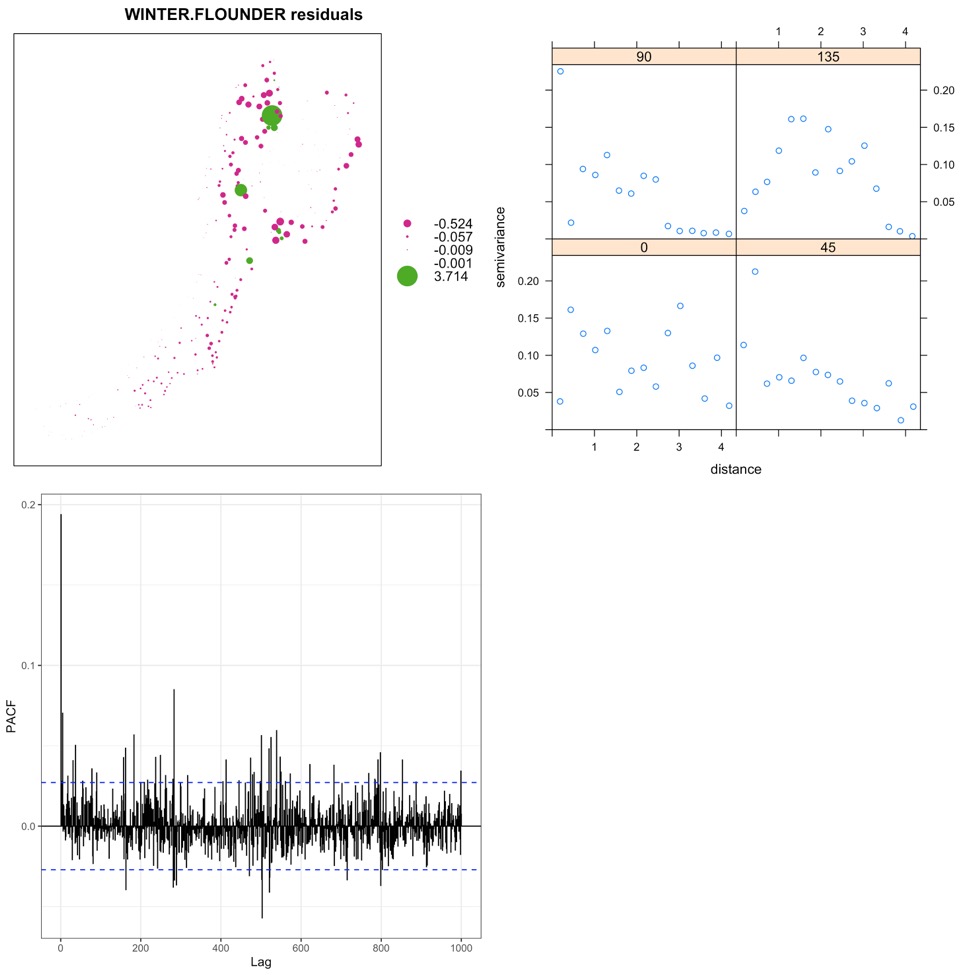

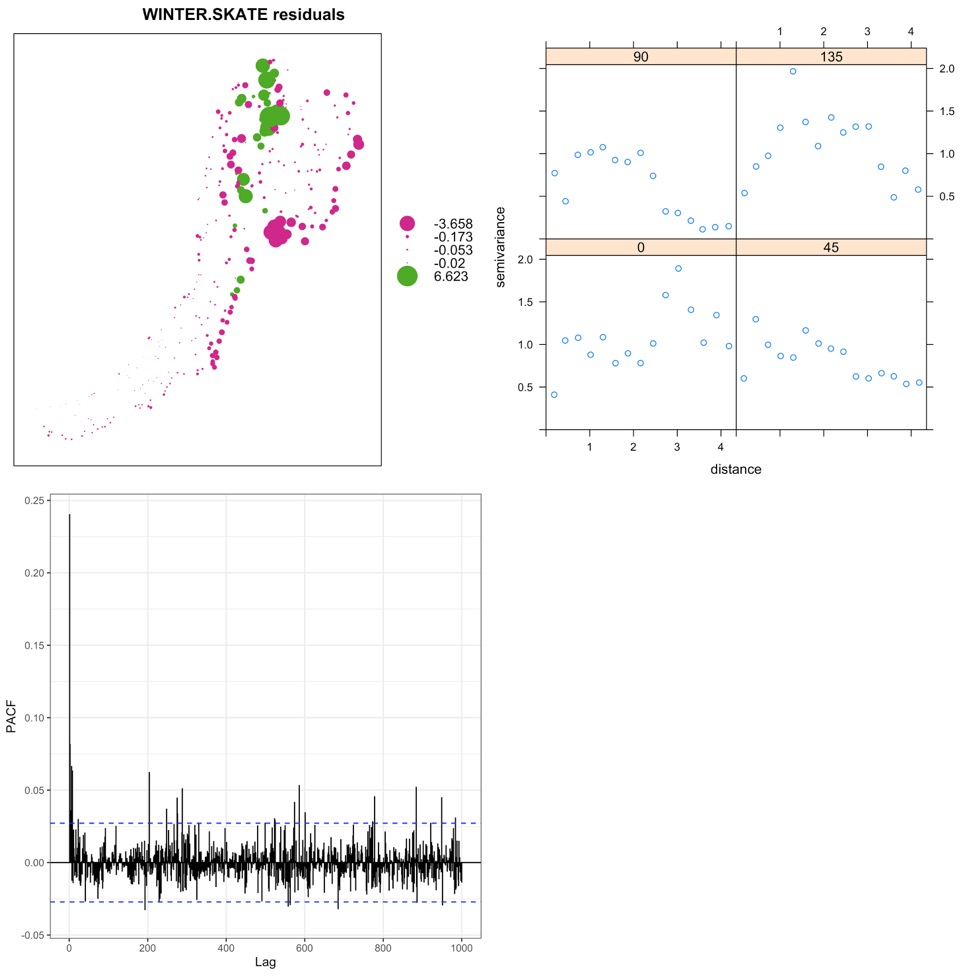

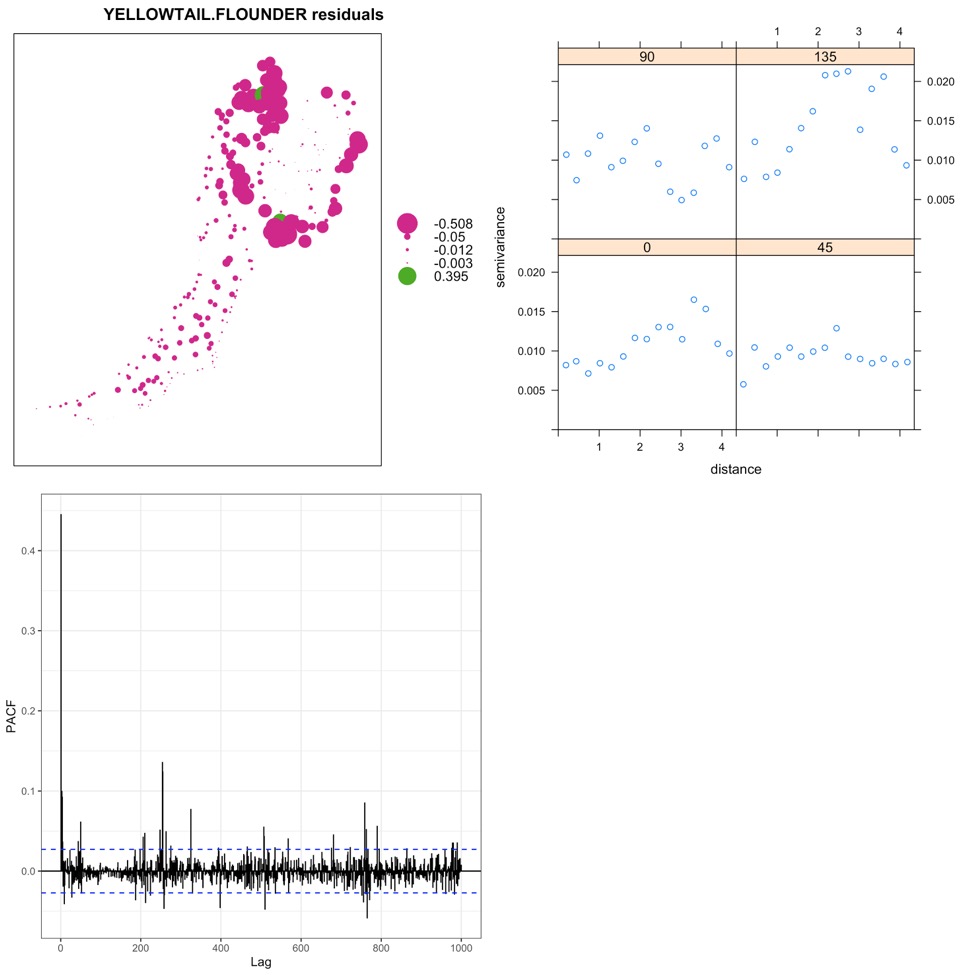

Supplement: Supplementary file 2 — Supplementary Figures. [file 41598_2021_4110_MOESM2_ESM.docx]
